# Phylogenetic Study of Local Patterns Influenza A(H3N2) Virus Transmission in a Semi‐Isolated Population in a Remote Island in Japan Between 2011 and 2013

**DOI:** 10.1111/irv.70089

**Published:** 2025-03-10

**Authors:** Su Myat Han, Teiichiro Shiino, Shingo Masuda, Yuki Furuse, Takahiro Yasaka, Satoshi Kanda, Kazuhiri Komori, Nobuo Saito, Yoshiano Kubo, Chris Smith, Akira Endo, Alexis Robert, Marc Baguelin, Koya Ariyoshi

**Affiliations:** ^1^ School of Tropical Medicine and Global Health Nagasaki University Nagasaki Japan; ^2^ Department of Infectious Disease Epidemiology, Faculty of Epidemiology and Population Health London School of Hygiene and Tropical Medicine London UK; ^3^ National Center for Infectious Disease Singapore; ^4^ Center for Clinical Sciences National Center for Global Health and Medicine Tokyo Japan; ^5^ AIDS Research Center National Institute of Infectious Diseases Tokyo Japan; ^6^ Department of Internal Medicine Kamigoto Hospital Kamigoto Japan; ^7^ Department of Medical Virology Nagasaki University Graduate School of Biomedical Sciences Nagasaki Japan; ^8^ Department of Microbiology, Faculty of Medicine Oita University Yufu Japan; ^9^ Department of Clinical Medicine, Institute of Tropical Medicine Nagasaki University Nagasaki Japan; ^10^ Department of Clinical Research, Faculty of Infectious and Tropical Diseases London School of Hygiene & Tropical Medicine London UK; ^11^ Centre for the Mathematical Modelling of Infectious Diseases London School of Hygiene & Tropical Medicine, Keppel Street London UK; ^12^ Saw Swee Hock School of Public Health National University of Singapore Singapore; ^13^ Infectious Disease Epidemiology and Dynamics, Institute of Tropical Medicine Nagasaki University Nagasaki Japan; ^14^ MRC Centre for Global Infectious Disease Analysis; and the Abdul Latif Jameel Institute for Disease London UK

**Keywords:** A/H3N2, influenza, molecular epidemiology, transmission dynamics

## Abstract

**Background:**

Influenza A outbreak risk is impacted by the potential for importation and local transmission. Reconstructing transmission history with phylogenetic analysis of genetic sequences can help assess outbreak risk but relies on regular collection of genetic sequences. Few influenza genetic sequences are collected in Japan, which makes phylogenetic analysis challenging, especially in rural, remote settings. We generated influenza A genetic sequences from nasopharyngeal swabs (NPS) samples collected using rapid influenza diagnostic tests and used them to analyze the transmission dynamics of influenza in a remote island in Japan.

**Methods:**

We generated 229 whole genome sequences of influenza A/H3N2 collected during 2011/12 and 2012/13 influenza seasons in Kamigoto Island, Japan, of which 178 sequences passed the quality check. We built time‐resolved phylogenetic trees from hemagglutinin sequences to classify the circulating clades by comparing the Kamigoto sequences to global sequences. Spatiotemporal transmission patterns were then analyzed for the largest local clusters.

**Results:**

Using a time‐resolved phylogenetic tree, we showed that the sequences clustered in six independent transmission groups (1 in 2011/12, 5 in 2012/13). Sequences were closely related to strains from mainland Japan. All 2011/12 strains were identified as clade 3C.2 (*n* = 29), while 2012/13 strains fell into two clades: clade 3C.2 (*n* = 129) and 3C.3a (*n* = 20). Clusters reported in 2012/13 circulated simultaneously in the same regions. The spatiotemporal analysis of the largest cluster revealed that while the first sequences were reported in the busiest district of Kamigoto, the later sequences were scattered across the island.

**Conclusion:**

Kamigoto Island was exposed to repeated importations of Influenza A(H3N2), mostly from mainland Japan, sometimes leading to local transmission and ultimately outbreaks. As independent groups of sequences overlapped in time and space, cases may be wrongly allocated to the same transmission group in the absence of genomic surveillance, thereby underestimating the risk of importations. Our analysis highlights how NPS could be used to better understand influenza transmission patterns in little‐studied settings and improve influenza surveillance in Japan.

## 1 Introduction

Seasonal influenza remains a major public health threat, causing substantial morbidity and mortality annually. It is estimated that influenza affects 3–5 million people globally, resulting in approximately 290,000–650,000 deaths each year [1]. The transmission patterns of seasonal influenza viruses have been extensively studied using epidemiological and genomic surveillance data [2, 3, 4, 5, 6], which helped understand influenza seasonal outbreak dynamics, identify the type of strain circulating at each season, and inform vaccine selection. It has also provided guidance on optimal timing for vaccination [3, 7, 8].

In Japan, the estimated annual prevalence of seasonal influenza exceeds 10% of the population [9]. Japan is home to many small islands, some of which have limited connections to mainland Japan [10], such as Kamigoto Island (Nagasaki Prefecture). In a previous study [2], we used influenza surveillance data on cases confirmed via rapid influenza diagnostic test (RIDT) in Kamigoto Island between 2010 and 2018 to identify determinants of transmission and local dynamics of influenza transmission. The study revealed patterns of transmission largely influenced by age, vaccine coverage, and district population density. However, surveillance data, based on onset dates, age groups, and residence of the cases, would not be able to distinguish between transmission chains cocirculating at the same time and areas, rendering accurate importation risk assessment challenging.

Phylogenetic analysis is instrumental in reconstructing the evolutionary relationship between genomic sequences of infected individuals sampled at different dates [11, 12, 13], and can help distinguish independent importations, and ultimately identify epidemiological relationship between cases. Cases with close genomic sequences are more likely to be epidemiologically connected, while different strains indicate separate importations. The time‐resolved phylogeny is also used to infer who‐infected‐whom among the sampled cases, thereby exploring the pathogen's transmission dynamics when the sampling density is high [11, 12, 14, 15, 16].

Since diverse Influenza A strains and subtypes can cocirculate, reliance solely on influenza‐like illness (ILI) and RIDT data often falls short of identifying distinct influenza strains. In Japan, a subset of the samples collected through RIDT are processed for subtype identification, but the size and representativeness of this subset are not sufficient to identify independent clusters and their origin or reconstruct transmission history. Genomic sequence data can bridge this knowledge gap, resulting in a deeper understanding of influenza's antigenic variability in a given setting, and their connections to other parts of the world.

In this study, we generated whole genome sequences (WGS) on reverse transcription‐polymerase chain reaction (RT‐PCR) using NPS samples from Kamigoto hospital routinely collected during the 2011/12 and 2012/13 influenza seasons. We made the sequences publicly accessible to improve the pool of available Japan influenza genetic sequences. We used these sequences and the date of collection of the cases to conduct phylogenetic analysis and understand the relationship between Kamigoto strains, mainland Japan strains, and the rest of the world. We assessed whether all cases were grouped in the same cluster or if they were related to independent importations and analyzed the role of importation and local transmission in the island. Finally, we describe the temporal and spatial distribution of the phylogenetic clusters. This analysis shows how NPS samples could be used for genomic surveillance in Japan, improving our understanding of influenza importation risk and spatial spread, and gives insights into influenza spread in rural settings.

## 2 Methods

### 2.1 Study Design and Setting

The samples used in this study were collected from patients recorded with ILI visiting to the Kamigoto Hospital in Kamigoto Island, Japan, during the 2011/12 and 2012/13 influenza seasons. Kamigoto Island is located in Nagasaki Prefecture, on the western coast of Japan, with a population of 22,599 inhabitants in 2011 [17]. Kamigoto Hospital is the only hospital in the island with all levels of care (primary, secondary, and tertiary care) and is part of the sentinel sites for influenza surveillance in Japan.

### 2.2 Sample Collection and WGS

The residual NPS from the RIDTs were temporarily stored at −20°C in the laboratory department of the hospital after being used for the RIDTs. The samples were transported to the Institute of Tropical Medicine at Nagasaki University within a week. The samples were stored in a deep freezer (−80°C) until further processed. Additional sociodemographic and clinical information on the patients was collected from the hospital database.

### 2.3 RNA Extraction and Influenza Virus Detection

Viral nucleic acid was extracted directly from the NPS samples using a QIAamp viral RNA mini kit (QIAGEN Inc., Valencia, CA) following the manufacturer's instructions. RNA was eluted to a final volume of 60 μL, aliquoted, and stored at −20°C for immediate use in reverse transcriptase‐polymerase chain reaction (RT‐PCR) or −80°C for long‐term storage. Multiplex RT‐PCR assays were applied to screen influenza viruses (A and B) and 11 other respiratory viruses. One‐Step RT‐PCR Kit (QIAGEN Inc., Valencia, CA, USA) was used for RNA viruses, and GoTaq Flexi DNA Polymerase (Promega, San Luis Obispo, CA, USA) and PCR Nucleotide Mix (Promega, San Luis Obispo, CA, USA) were used for DNA viruses. The details of the multiplex PCR assay protocols were described elsewhere [18].

### 2.4 Multisegment RT‐PCR, Library Preparation, and Next‐Generation Sequencing (NGS)

For all RT‐PCR‐confirmed influenza A positive samples, all eight segments of RT‐PCR‐confirmed influenza samples were amplified following the protocols from Zhou, B., et al. (2009) [19] for influenza A. Each PCR product was purified again using Ampure XP beads (Beckman Coulter) according to the manufacturer's instructions. The purity was then assessed with Agilent Technology 2100 Bioanalyzer using a High Sensitivity DNA chip and Qubit dsDNA HS Assay Kit (Life Technology). One nanogram of the DNA was used for library preparation (Nextera XT Kit, Illumina) following the manufacturer's instructions (Illumina, CA, USA). The prepared library was sequenced on the MiSeq platform (Illumina) using a V2 2 × 250‐bp reagents kit at Nagasaki University.

### 2.5 NGS Data Processing and Genome Assembly

A total of 254 samples were available for WGS. The sequencing output was uploaded to the INSaFLU [20], a web‐based bioinformatic platform for influenza sequencing analysis. By providing raw sequence data, INSaFLU performs (i) quality check (using FastQC) [21] and (ii) removal of adapter sequences and low‐quality reads (using Trimmomatic) [22]. Trimmed and filtered sequences of less than 100 bp were discarded in downstream analysis. The sequence reads were mapped to reference sequence *A/H3N2_A_Perth_16_2009*. Consensus for sequences with at least tenfold coverage was then generated. The INSAFLU also performed the genome annotation (using Prokka) [23].

### 2.6 Phylogenetic Analysis

To contextualize the Kamigoto samples within the landscape of viruses circulating in Japan and globally during the 2011/12 and 2012/13 influenza seasons, we downloaded A/H3N2 influenza sequences from GISAID (https://www.gisaid.org) sampled between January 1, 2011, and December 31, 2013 for each individual segment (PB2, PB1, PA, HA, NP, NA, MP, and NS) (as of January 1, 2023). For each individual segment, sequence alignment was performed with MAFFT version 7 [23], and the alignments were visualized and manually edited in AliView [24].

Phylogenetic trees of each segment data were estimated using the maximum‐likelihood (ML) procedure in IQTREE v2.0.7 [25], under the best‐fitting model of nucleotide substitution TVM + F + I + I + R5, as determined by the ModelFinder option implemented in software. Branch supports were estimated by standard nonparametric bootstrap analysis with 100 replicates. Clades were labelled following WHO nomenclature. TempEst v1.5.3 [26] was used to assess the presence of a molecular clock signal in the analyzed data, and linear regression of root‐to‐tip genetic distances against sampling dates was reconstructed (Figure S1). Outlier sequences that significantly deviated from the mean likely contain sequencing artifacts and were excluded from further analysis. We performed time‐scaled phylogenies of final hemagglutinin (HA) segment and root‐to‐tip linear regressions using TreeTime [27] based on the ML trees generated by IQTREE. The spatial distribution of the sequences included in this analysis was: Africa (*n* = 39), Asia (*n* = 180, including 90 sequences from Japan), Europe (*n* = 57), North America (*n* = 365), Oceania (*n* = 111), and South America (*n* = 82).

We also downloaded all publicly available full‐length sequences from Japan for all eight influenza segments from the GISAID [28] (*n* = 88, as of April 2020). All eight influenza A(H3N2) coding sequences in the sequences downloaded from Japan and Kamigoto were concatenated into an alignment of 13,136 nucleotides in the order of the segment number (PB2‐PB1‐PA‐HA‐NP‐NA‐M‐NS). The concatenated sequences were aligned using MAFFT version 7 [23] and the alignments were visualized and manually edited in AliView [24]. ML phylogenetic trees were reconstructed for the WGS of Japan and Kamigoto strains, as mentioned above.

## 3 Results

### 3.1 WGS of A/H3N2 Samples

**FIGURE 1:**
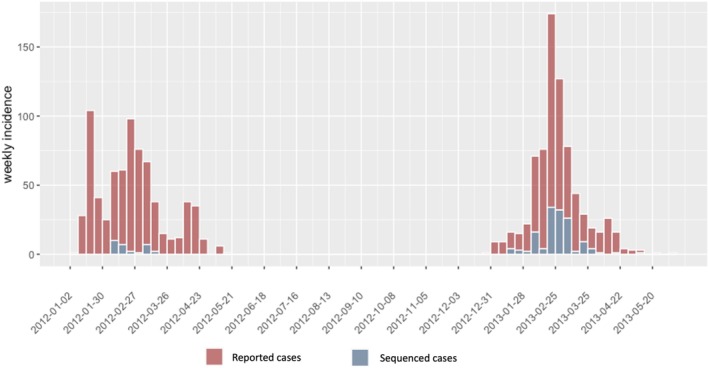
Epidemic curve of the daily number of rapid influenza diagnostic test‐positive cases reported by the hospital influenza surveillance system (red) and whole genome sequenced (WGS) cases (blue) during the 2011/12 and 2012/13 epidemic seasons.

Figure 1 displays the epidemiological curve aggregated by reported RIDT positive cases and sequenced cases. A total of 2316 nasopharyngeal swab samples from ILI cases were collected across Kamigoto, during the study seasons. Of these, 538 samples were confirmed as influenza A positive by multiplex RT‐PCR. Out of 538 influenza A positive samples, 254 samples were available for WGS. A further 25 sequences were discarded as the subtype was not available. We generated 229 WGS characterized as A(H3N2) (29 samples from the 2011/12 season and 200 from the 2012/13 season). Out of the 229 WGS, 178 achieved 100% coverage for by at least onefold for the HA segment, and 166 achieved 100% of sizes covered by at least onefold for all segments (see Figure S2 for a description of the sample selection process). The overview of the depth and length sequence coverage for each segment for all samples is provided in Figure S3.

### 3.2 Phylogenetic Analysis of Sequences

**FIGURE 2:**
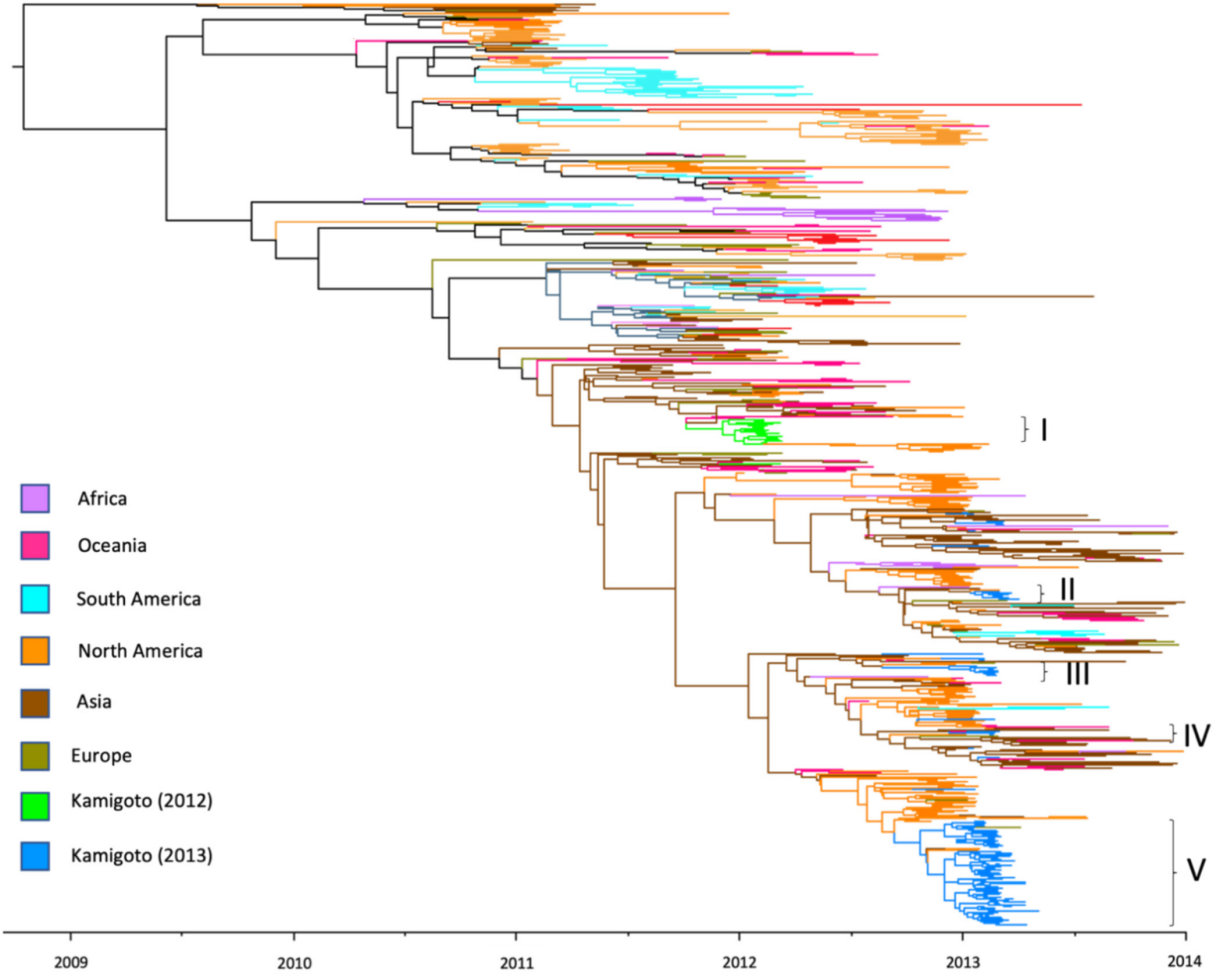
Time‐scaled maximum‐likelihood phylogenetic trees of HA coding sequences for A(H3N2) viruses circulating in Kamigoto and comparing sequences from strains isolated in Japan and other parts of the world from GISAID collected between 2011 and 2013. (as of December 1, 2022 as submission date). All full‐length HA coding gene sequences were downloaded from GISAID, with duplicate sequences removed. Colored by geographical region.

We assessed how the HA segments of the 178 WGS from Kamigoto compared to publicly available sequences from GISAID sampled globally between January 2011 and December 2013 by inferring a phylogenetic tree using the HA sequences (Figures 2 and S4). Smaller, closely related subgroups were identified within the seasons. ML phylogenetic analysis of the HA segment for Kamigoto strains revealed the predominant circulation of clade 3C.2 on the island during the 2011/12 and 2012/13 seasons. However, a few Kamigoto sequences (20) from the 2012/13 season were classified under clade 3C.3a. (Figure S5).

Most Kamigoto sequences clustered in six independent transmission groups (Figure 2), with sequences in Group 5 further classified as 5a (28 sequences) and 5b (81 sequences), as these sequences are closer than other groups, but their date of coalescence is too far from their onset date of the cases, suggesting different introductions (Figures 2 and S4). The phylogeny identified that the Kamigoto sequences were closely related to strains from mainland Japan, and local sequences in each group were observed to be closely clustered with strains from mainland Japan. The biggest group (Group 5) consists of 109 local sequences and 21 nonlocal sequences from mainland Japan (*n* = 13), North America (*n* = 6), Europe (*n* = 1), and Asia (*n* = 1). The second biggest group (Group 1) was from the 2011/12 season, which consisted of 27 local sequences and 33 nonlocal sequences from mainland Japan (*n* = 10), North America (*n* = 12), Europe (*n* = 3), Oceania (n = 3), and Asia (*n* = 5). The group 2 consists of 6 Kamigoto sequences and Group 3 consists of 10 Kamigoto sequences. The group 4 includes 11 Kamigoto sequences, and 5 sequences from mainland Japan. The clustering of a large number of sequences on the phylogeny suggested that the majority of the cases sequenced in this study are likely to be part of different local transmission chains.

A few Kamigoto sequences (*n* = 7) were isolated, without clustering with any other Kamigoto sequences. These sequences formed isolated branches on the phylogenetic tree, separate from the main clusters associated with local transmission. This pattern suggests that these sequences may represent independent introductions of the virus to Kamigoto rather than ongoing local circulation. It is possible that subsequent transmission for these outliers either did not occur or cases arising from them were not available for sequencing to be included in this study.

We also constructed phylogenetic trees of the other individual segments of Kamigoto strains (PB2, PB1, PA, NP, NA, MP, and NS), with publicly available strains from Japan and global sequences from GISAID as of the December 1, 2022, submission date (Figures S6–S13). We separately performed the phylogenetic analysis for each segment to avoid the reassortment bias between the segments. The findings from these segments were consistent with the phylogenetic analysis of the HA segment.

The ML phylogeny of WGS of A/H3N2 from Kamigoto Island (Figure S14) also showed that Kamigoto sequences grouped closely together based on the year they were collected. No sequence from the 2011/12 season grouped with the 2012/13 sequences.

### 3.3 Epidemiological Description of the Transmission Clusters

**FIGURE 3:**
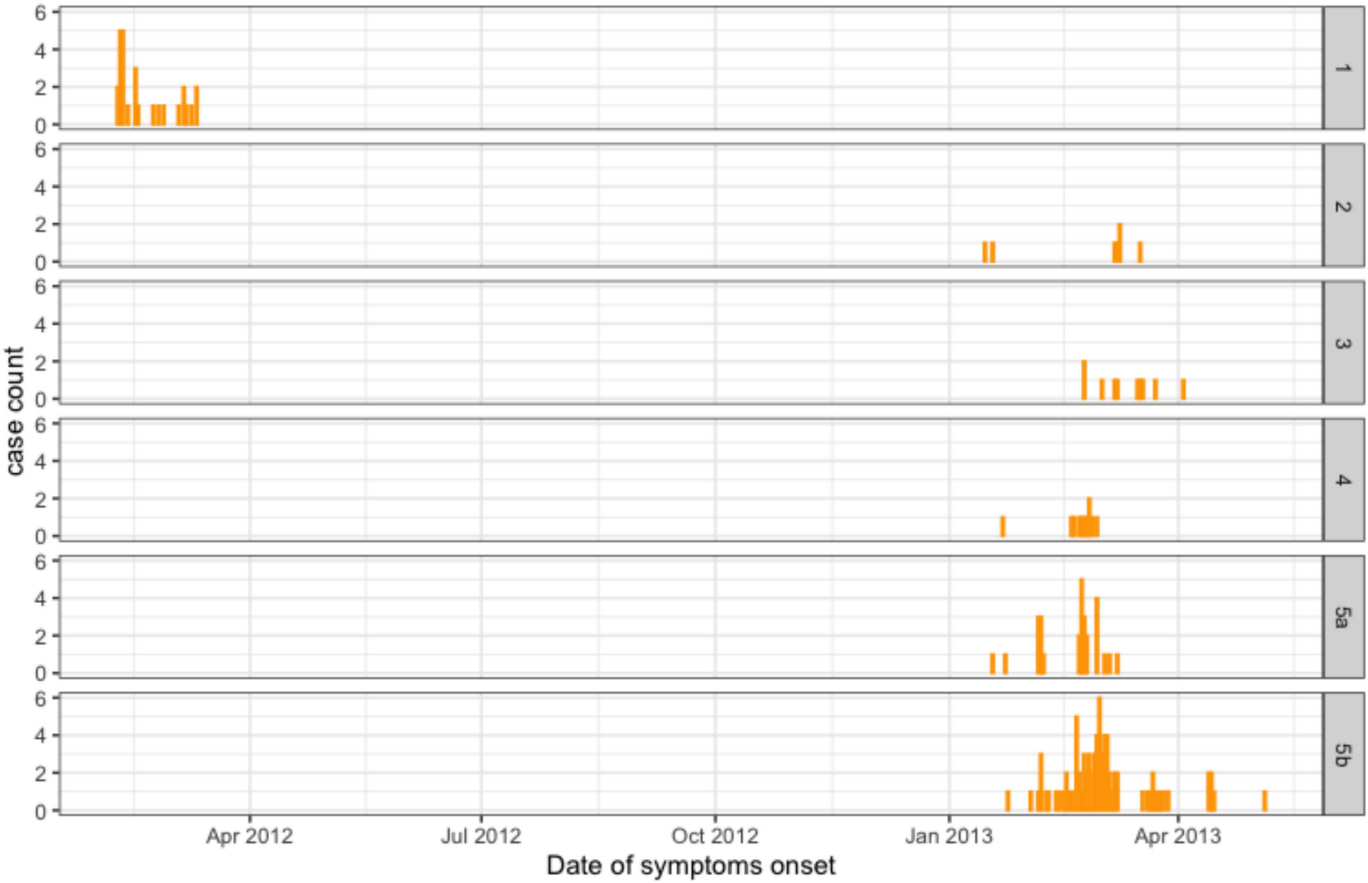
Clusters distribution from time‐resolved phylogenetic tree of HA segments of A(H3N2) viruses from Kamigoto Island, Japan.

**FIGURE 4:**
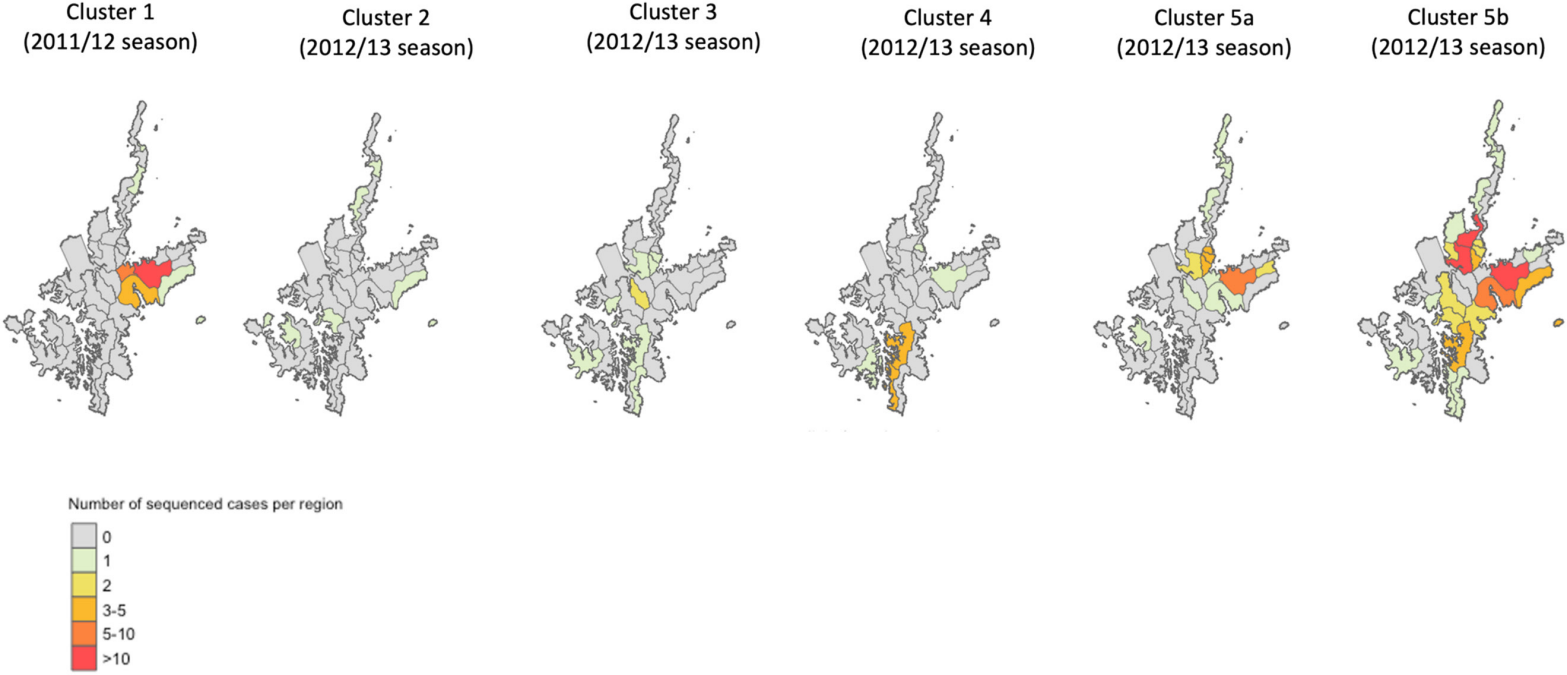
Geographic distribution of the clusters identified during 2011/12 and 2012/13 influenza seasons.

Kamigoto sequences collected in 2012/13 clustered in five groups, which cocirculated during the same period, mainly between February and April 2013 (Figure 3). This suggested that different importation events initiated the transmission and further drove local transmission. We then explored the geographic distribution of the Kamigoto strains within these groups (Figure 4). Group 3, Group 4, and Group 5a had overlapping locations of the sequenced cases, while Group 2 was geographically isolated. The biggest local Kamigoto sequence Group 5b was found to be distributed across the island (Figure 4).

**FIGURE 5:**
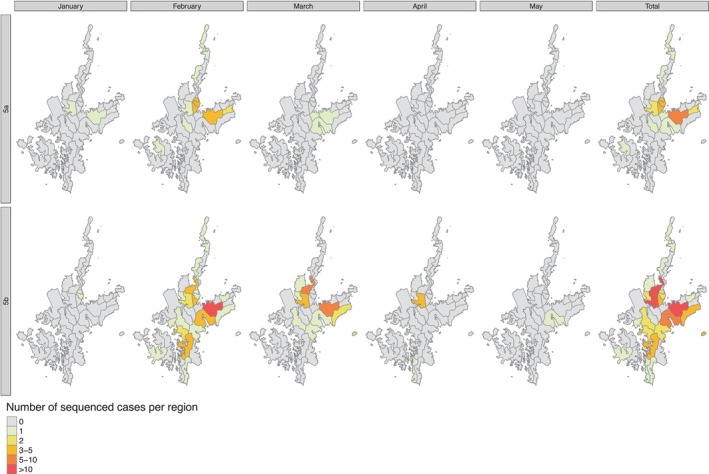
Spatiotemporal distribution of the Group 5a and 5b.

We further analyzed the biggest transmission group of Kamigoto sequences (Group 5a and 5b) to explore the spatiotemporal distribution patterns (Figures 5 and S15). The spatiotemporal assessment of the clusters showed the virus spreading from populous districts to less populous ones and between busier or more populous districts, such as Aogata‐go (an urban area of the island) and Tainoura‐go and Arikawa‐go (port areas connecting to mainland Japan). The earliest sequenced case of Group 5a was reported on January 18, 2013. The number of cases increased in subsequent weeks in the same districts and further spread out to other districts in February and March. The earliest sequenced case of Group 5b was reported in January 24, 2013 and the highest number of cases from this cluster was reported in February 2013. The outbreaks slowly declined from March onwards in both groups. The last sequenced case was reported on the March 8, 2013 in Group 5a and May 5, 2013 in Group. RT‐PCR confirmed influenza‐positive surveillance data (Figure 1) also showed February to April as the peak of the season. A similar temporospatial distribution was also observed in local sequences cluster in Group 1 (Figure S16).

## 4 Discussion

In this study, we conducted a retrospective genomic characterization of seasonal influenza A/H3N2 viruses collected from Kamigoto Island, Japan, during 2011/12 and 2012/13 influenza seasons. We extracted RNA from NPS samples and successfully sequenced and generated WGS of 229 A/H3N2 samples: 29 from the 2011/12 season and 200 from the 2012/13 season. This contribution is particularly valuable, as there are limited WGS available from Japan for that time period: 88 sequences between 1968 and 2020, but less than 10 in 2011/12 and zero in 2012/13 influenza season (as of March 2022). Given the increasing importance of genomic surveillance in monitoring and controlling influenza outbreaks, this study provides crucial data that enhances our understanding of the virus's evolution and spread within Japan during these seasons.

In our study, WGS of all influenza A‐positive samples identified the viruses as A/H3N2 for both the 2011/12 and 2012/13 influenza seasons. This finding is consistent with a study conducted in Okinawa [24], where viral cultures from the same seasons also identified A/H3N2 exclusively. Additionally, a national report from the National Institute of Infectious Diseases (NIID), Japan, indicated that A/H3N2 was the dominant strain during these seasons [25, 26]. Furthermore, the World Health Organization (WHO) Western Pacific Region also reported the dominance of A/H3N2 during this period [27]. These consistent findings across multiple sources highlight the predominance of A/H3N2 in the study period.

Using the time‐resolved phylogeny, we identified multiple independent importations of A(H3N2) viruses into the Kamigoto Island community during the study period, some of these importations led to local transmission. Kamigoto's geographical isolation and reliance on two ports for travel create distinct conditions for the introduction and transmission of influenza viruses. Unlike urban areas with continuous high population movement, the patterns observed in Kamigoto suggest episodic importation of influenza strains from mainland Japan, likely through seasonal travel. The close genetic relationship between Kamigoto strains and those from mainland Japan highlights the importance of monitoring travel‐associated importations in shaping local outbreaks. This finding contrasts with findings from studies conducted in Kilifi County, Kenya [29], and Basel city in Switzerland [30]. In those studies, strains from Kenya and Basel displayed close genetic relations to global strains, reflecting the high connectivity of these urban centers with other parts of the world. Such differences highlight the significance of understanding influenza dynamics at the local or rural level since transmission patterns can vary based on geographical factors. However, due to the limited availability of local‐level studies in Japan and globally, comparing trends remains challenging. Notably, few studies focus on transmission dynamics in specific settings like cities, towns, or islands [29, 30]. Most research investigates global, regional, or country‐level transmission, or, conversely, is limited to localized environments such as universities, schools, or camps [28, 31, 32]. Interestingly, our study revealed a few potential international importation events where the Kamigoto strains closely resembled those from outside Japan in the phylogenetic analysis. This underscores the importance of regularly testing visitors and returning residents, especially during influenza season. Low density of sequence numbers publicly available from the southern part of Japan during the same period may also be another reason explaining the close relatedness of the Kamigoto strains to outside Japan.

We found Kamigoto strains clustered in six different groups of sequences in the phylogenetic tree. Notably, Kamigoto sequence clusters in 2012/13 were cocirculating during the same period (between February and April 2013), indicating that different importation events fueled the transmission of influenza in each group. In the absence of genomic surveillance, cases circulating simultaneously may be inaccurately classified into the same cluster using only epidemiological data, which would underestimate the importation risks, and potentially overestimate the risk of local outbreak following importations. This highlights the value of genomic surveillance, particularly in the context of pandemic preparedness, as it enables a deeper understanding of viral relationships and transmission pathways beyond localized outbreaks. Our previous study of influenza outbreaks in Kamigoto over an 8‐year period [ 2], based on RIDT surveillance data, was able to explore transmission patterns within the islands but could not detect connections to strains beyond Kamigoto. The finding of the spatial distribution of the sequences in Group 5b align with our earlier epidemiological study, which identified districts with connections outside the island and high population density as the main drivers of influenza transmission patterns at the local community level.

Although the study used samples that were collected more than 9 years ago, a good viral yield was retrieved by direct RNA extraction from the samples. This is a benefit of sequencing directly from the samples (rather than passaging or isolation) and storage with optimal temperature for a long duration. Only few sequences were available from the Kyuushuu regions (where Kamigoto is located) prior to this project. The sequences collected, shared, and analyzed in this paper, therefore, greatly improve our understanding and knowledge of influenza phylogeny in Japan. In Japan, RIDT was widely used during the influenza season, and the residual samples from NPS can be utilized for genomic surveillance to describe the genetic diversity of circulating influenza A strains. This nationwide data provides a valuable resource for understanding viral evolution and informing public health strategies.

The sequenced samples were dependent on the sample viral load and cDNA concentration to pass the sequencing quality and thus it limits the representativeness of the data in the reconstruction of the influenza transmission. However, the sequenced samples were roughly proportional to the reported RIDT‐confirmed influenza cases. The number of sequences generated in this study were relatively low compared to the reported number of influenza cases in that period (1009 and 1053 RIDT cases were reported in 2011/12 and 2012/13, Figure S1). An increased sequencing frequency may capture more introduction events and clusters. Finally, the study was part of the surveillance system, and thus data were collected during the influenza season (October–June) rather than the whole year. This limits our study's assessment of year‐round influenza transmission patterns within the islands.

## 5 Conclusion

This analysis helps to better understand the transmission patterns of seasonal influenza because it was conducted in rural setting such as Kamigoto Island with a semi‐isolated population in Japan. Influenza A(H3N2) virus epidemics in Kamigoto Island were marked by multiple introductions and fueled by local transmission. A closer examination of local transmission through genomic data indicates concurrent independent introduction events and local proliferation. These might be misinterpreted as part of the same cluster without sequencing data. However, the results of this study are based on a 2‐year analysis of influenza sequences from the island; thus, repeated analyses for different influenza seasons and geographic locations will help us better understand detailed transmission patterns.

## Author Contributions

**Su Myat Han:** conceptualization, data curation, formal analysis, writing – original draft, visualization, writing – review and editing. **Teiichiro Shiino:** data curation, software, validation, visualization, writing – review and editing. **Shingo Masuda:** resources, investigation. **Yuki Furuse:** methodology, writing – review and editing. **Takahiro Yasaka:** resources, investigation. **Satoshi Kanda:** resources, investigation. **Kazuhiri Komori:** investigation, resources. **Nobuo Saito:** investigation, resources. **Yoshiano Kubo:** validation, visualization. **Chris Smith:** funding acquisition, writing – review and editing. **Akira Endo:** conceptualization, writing – review and editing. **Alexis Robert:** formal analysis, methodology, supervision, writing – review and editing. **Marc Baguelin:** project administration, funding acquisition, supervision, resources, writing – review and editing. **Koya Ariyoshi:** funding acquisition, writing – review and editing, project administration, supervision, resources.

## Ethics Statement

The research was approved by the institutional review boards of Kamigoto Hospital, Nagasaki University Research Ethics Committee (reference number 200619236), and the London School of Hygiene and Tropical Medicine Research Ethics Committee (reference number 26706). Both Nagasaki University and London School of Hygiene and Tropical Medicine granted waivers for obtaining informed consent due to the nature of this retrospective study and the preserved anonymity of patients.

## Conflicts of Interest

The authors declare no conflicts of interest.

### Peer Review

The peer review history for this article is available at https://www.webofscience.com/api/gateway/wos/peer‐review/10.1111/irv.70089.

## Supporting information


**Figure S1.** Regression of root‐to‐tip genetic distances against sample collection dates.
**Figure S2.** Flowchart showing from samples collection to whole genome sequencing (WGS). Twenty‐five of the 254 samples available for WGS were discarded as subtype was not available.
**Figure S3.** Boxplots illustrating median read depth across each segment for A/H3N2 virus. Boxes extend to the 1st and 3rd quartile.
**Figure S4.** Time‐resolved phylogenetic tree of HA segments of Kamigoto Island, Japan and global sequences (GISAID) as of December 1, 2022 submission date.
**Figure S5.** Maximum‐likelihood phylogenetic tree of HA segments of A(H3N2) viruses from Kamigoto Island, Japan. The sequences are colored by season (red 2011/12, blue 2012/2023).
**Figure S6.** Maximum‐likelihood phylogenetic trees of PB2 segments of influenza A/H3N2 viruses circulating in Kamigoto and comparing sequences from strains isolated in Japan and other parts of the world from GISAID collected between 2011 and 2013.Kamigoto sequences are in red color. The remaining strains are colored coded by region: North America in cyan, South America in teal, Oceania in green, Africa in magenta, Europe in purple, and Asia in brown.
**Figure S7.** Maximum‐likelihood phylogenetic trees of PB1 segments of influenza A/H3N2 viruses circulating in Kamigoto and comparing sequences from strains isolated in Japan and other parts of the world from GISAID collected between 2011 and 2013. Kamigoto sequences are in red color. The remaining strains are colored coded by region: North America in cyan, South America in teal, Oceania in green, Africa in magenta, Europe in purple, and Asia in brown.
**Figure S8.** Maximum‐likelihood phylogenetic trees of PA segments of influenza A/H3N2 viruses circulating in Kamigoto and comparing sequences from strains isolated in Japan and other parts of the world from GISAID collected between 2011 and 2013. Kamigoto sequences are in red color. The remaining strains are colored coded by region: North America in cyan, South America in teal, Oceania in green, Africa in magenta, Europe in purple, and Asia in brown.
**Figure S9.** Maximum‐likelihood phylogenetic trees of HA segments of influenza A/H3N2 viruses circulating in Kamigoto and comparing sequences from strains isolated in Japan and other parts of the world from GISAID collected between 2011 and 2013. Kamigoto sequences are in red color. The remaining strains are colored coded by region: North America in cyan, South America in teal, Oceania in green, Africa in magenta, Europe in purple, and Asia in brown.
**Figure S10.** Maximum‐likelihood phylogenetic trees of NP segments of influenza A/H3N2 viruses circulating in Kamigoto and comparing sequences from strains isolated in Japan and other parts of the world from GISAID collected between 2011 and 2013. Kamigoto sequences are in red color. The remaining strains are colored coded by region: North America in cyan, South America in teal, Oceania in green, Africa in magenta, Europe in purple, and Asia in brown.
**Figure S11.** Maximum‐likelihood phylogenetic trees of NA segments of influenza A/H3N2 viruses circulating in Kamigoto and comparing sequences from strains isolated in Japan and other parts of the world from GISAID collected between 2011 and 2013. Kamigoto sequences are in red color. The remaining strains are colored coded by region: North America in cyan, South America in teal, Oceania in green, Africa in magenta, Europe in purple, and Asia in brown.
**Figure S12.** Maximum‐likelihood phylogenetic trees of MP segments of influenza A/H3N2 viruses circulating in Kamigoto and comparing sequences from strains isolated in Japan and other parts of the world from GISAID collected between 2011 and 2013. Kamigoto sequences are in red color. The remaining strains are colored coded by region: North America in cyan, South America in teal, Oceania in green, Africa in magenta, Europe in purple, and Asia in brown.
**Figure S13.** Maximum‐likelihood phylogenetic trees of NS segments of influenza A/H3N2 viruses circulating in Kamigoto and comparing sequences from strains isolated in Japan and other parts of the world from GISAID collected between 2011 and 2013. Kamigoto sequences are in red color. The remaining strains are colored coded by region: North America in cyan, South America in teal, Oceania in green, Africa in magenta, Europe in purple, and Asia in brown.
**Figure S14.** The maximum‐likelihood phylogenetic tree includes 166 WGS collected during the 2011/12 (red) and 2012/13 (purple) influenza seasons alongside the WGS in Japan, that were available in the GISAID (blue), and the vaccine strains (green). For each sequence, the date of the sample collection is mentioned (yyyy‐mm‐dd). WGS for 2012/2013 in Japan were not available during the study period.
**Figure S15.** Temporal and spatial distribution of the sequenced cases of Cluster 5 (5A and 5B) (weekly).
**Figure S16.** Temporal and spatial distribution of the sequenced cases of Cluster 1 (weekly).
**Table S1.** GISAID accession number of sequences produced in this study.

## Data Availability

All consensus sequences we generated have been submitted to the GISAID database. The details of isolate IDs and assigned accession numbers are listed in Table S1.
