# Supplementary material for: Phylogenetic Study of Local Patterns Influenza A(H3N2) Virus Transmission in a Semi‐Isolated Population in a Remote Island in Japan Between 2011 and 2013
Source: Influenza Other Respir Viruses. 2025 Mar 10;19(3):e70089. doi: 10.1111/irv.70089 (PMC11893481; doi:10.1111/irv.70089)
Supplement: Supplementary file 1 — Figure S1. Regression of root‐to‐tip genetic distances against sample collection dates. Figure S2. Flowchart showing from samples collection to whole genome sequencing (WGS). Twenty‐five of the 254 samples available for WGS were discarded as subtype was not available. Figure S3. Boxplots illustrating median read depth across each segment for A/H3N2 virus. Boxes extend to the 1st and 3rd quartile. Figure S4. Time‐resolved phylogenetic tree of HA segments of Kamigoto Island, Japan and global sequences (GISAID) as of December 1, 2022 submission date. Figure S5. Maximum‐likelihood phylogenetic tree of HA segments of A(H3N2) viruses from Kamigoto Island, Japan. The sequences are colored by season (red 2011/12, blue 2012/2023). Figure S6. Maximum‐likelihood phylogenetic trees of PB2 segments of influenza A/H3N2 viruses circulating in Kamigoto and comparing sequences from strains isolated in Japan and other parts of the world from GISAID collected between 2011 and 2013.Kamigoto sequences are in red color. The remaining strains are colored coded by region: North America in cyan, South America in teal, Oceania in green, Africa in magenta, Europe in purple, and Asia in brown. Figure S7. Maximum‐likelihood phylogenetic trees of PB1 segments of influenza A/H3N2 viruses circulating in Kamigoto and comparing sequences from strains isolated in Japan and other parts of the world from GISAID collected between 2011 and 2013. Kamigoto sequences are in red color. The remaining strains are colored coded by region: North America in cyan, South America in teal, Oceania in green, Africa in magenta, Europe in purple, and Asia in brown. Figure S8. Maximum‐likelihood phylogenetic trees of PA segments of influenza A/H3N2 viruses circulating in Kamigoto and comparing sequences from strains isolated in Japan and other parts of the world from GISAID collected between 2011 and 2013. Kamigoto sequences are in red color. The remaining strains are colored coded by region: North America in cyan, South [file IRV-19-e70089-s001.docx]

Phylogenetic study of local patterns influenza A(H3N2) virus transmission in a semi-isolated population in a remote island in Japan between 2011-2013

Su Myat Han^1,2,3^, Teiichiro Shiino^4,5^, Shingo Masuda^1,6^, Yuki Furuse^7^, Takahiro Yasaka^6^, Satoshi Kanda^6^, Kazuhiri Komori^6^, Nobuo Saito^1,8^, Yoshiano Kubo^9^, Chris Smith^1,10^, Akira Endo^1,2,11,12^, Alexis Robert^1,2,11*^, Marc Baguelin^2,11,13*^, Koya Ariyoshi^1,9*^

1. School of Tropical Medicine and Global Health, Nagasaki University, Nagasaki, Japan
2. Department of Infectious Disease Epidemiology, Faculty of Epidemiology and Population Health, London School of Hygiene and Tropical Medicine, London, United Kingdom
3. National Center for Infectious Disease, Singapore
4. Center for Clinical Sciences, National Center for Global Health and Medicine, Tokyo, Japan
5. AIDS Research Center, National Institute of Infectious Diseases, Tokyo, Japan
6. Department of Internal Medicine, Kamigoto Hospital, Kamigoto, Japan
7. Department of Medical Virology, Nagasaki University Graduate School of Biomedical Sciences, Nagasaki, Japan
8. Department of Microbiology, Faculty of Medicine, Oita University, Yufu, Japan
9. Department of Clinical Medicine, Institute of Tropical Medicine, Nagasaki University, Nagasaki, Japan
10. Department of Clinical Research, Faculty of Infectious and Tropical Diseases, London School of Hygiene & Tropical Medicine, London, UK
11. Centre for the Mathematical Modelling of Infectious Diseases, London School of Hygiene & Tropical Medicine, Keppel Street, London, UK
12. Saw Swee Hock School of Public Health, National University of Singapore
13. MRC Centre for Global Infectious Disease Analysis; and the Abdul Latif Jameel Institute for Disease

*****  **These authors are joint last authors**

Table S-1: GISAID accession number of sequences produced in this study.

|  | Isolate_Name | Isolate_Id | PB2 | PB1 | PA | HA | NP | NA_Seg | MP | NS |
| --- | --- | --- | --- | --- | --- | --- | --- | --- | --- | --- |
| 1 | A/Kamigoto/728/2012 | EPI_ISL_17103063 | EPI2449327 | EPI2449328 | EPI2449329 | EPI2449330 | EPI2449331 | EPI2449332 | EPI2449333 | EPI2449334 |
| 2 | A/Kamigoto/199/2013 | EPI_ISL_17092317 | EPI2440111 | EPI2440227 | EPI3785003 | EPI2440229 | EPI2440230 | EPI2440231 | EPI2440232 | EPI2440233 |
| 3 | A/Kamigoto/1454/2013 | EPI_ISL_18002450 | EPI2635343 | EPI2635344 | EPI2635345 | EPI2635346 | EPI2635347 | EPI2635348 | EPI2635349 | EPI2635350 |
| 4 | A/Kamigoto/1461/2013 | EPI_ISL_18002449 | EPI2635335 | EPI2635336 | EPI3785002 | EPI2635338 | EPI2635339 | EPI2635340 | EPI2635341 | EPI2635342 |
| 5 | A/Kamigoto/1444/2013 | EPI_ISL_18002434 | EPI2635327 | EPI2635328 | EPI2635329 | EPI2635330 | EPI2635331 | EPI2635332 | EPI2635333 | EPI2635334 |
| 6 | A/Kamigoto/1467/2013 | EPI_ISL_18002433 | EPI2635319 | EPI2635320 | EPI2635321 | EPI2635322 | EPI2635323 | EPI2635324 | EPI2635325 | EPI2635326 |
| 7 | A/Kamigoto/1442/2013 | EPI_ISL_18002432 | EPI2635311 | EPI2635312 | EPI2635313 | EPI2635314 | EPI2635315 | EPI2635316 | EPI2635317 | EPI2635318 |
| 8 | A/Kamigoto/1476/2013 | EPI_ISL_18001909 | EPI2635303 | EPI2635304 | EPI2635305 | EPI2635306 | EPI2635307 | EPI2635308 | EPI2635309 | EPI2635310 |
| 9 | A/Kamigoto/1480/2013 | EPI_ISL_18001908 | EPI2635295 | EPI2635296 | EPI2635297 | EPI2635298 | EPI2635299 | EPI2635300 | EPI2635301 | EPI2635302 |
| 10 | A/Kamigoto/1437/2013 | EPI_ISL_18001895 | EPI2635239 | EPI2635240 | EPI2635241 | EPI2635242 | EPI2635253 | EPI2635265 | EPI2635278 | EPI2635283 |
| 11 | A/Kamigoto/1433/2013 | EPI_ISL_18001894 | EPI2635231 | EPI2635232 | EPI2635233 | EPI2635234 | EPI2635235 | EPI2635236 | EPI2635237 | EPI2635238 |
| 12 | A/Kamigoto/1432/2013 | EPI_ISL_18001808 | EPI2635135 | EPI2635136 | EPI2635146 | EPI2635161 | EPI2635174 | EPI2635186 | EPI2635197 | EPI2635211 |
| 13 | A/Kamigoto/1431/2013 | EPI_ISL_18001804 | EPI2635127 | EPI2635128 | EPI2635129 | EPI2635130 | EPI2635131 | EPI2635132 | EPI2635133 | EPI2635134 |
| 14 | A/Kamigoto/1420/2013 | EPI_ISL_18001803 | EPI2635119 | EPI2635120 | EPI2635121 | EPI2635122 | EPI2635123 | EPI2635124 | EPI2635125 | EPI2635126 |
| 15 | A/Kamigoto/1482/2013 | EPI_ISL_18001802 | EPI2635111 | EPI2635112 | EPI2635113 | EPI2635114 | EPI2635115 | EPI2635116 | EPI2635117 | EPI2635118 |
| 16 | A/Kamigoto/1491/2013 | EPI_ISL_18001801 | EPI2635076 | EPI2635094 | EPI2635105 | EPI2635106 | EPI2635107 | EPI2635108 | EPI2635109 | EPI2635110 |
| 17 | A/Kamigoto/1494/2013 | EPI_ISL_18001747 | EPI2634970 | EPI2634971 | EPI2634972 | EPI2634973 | EPI2634974 | EPI2634975 | EPI2634976 | EPI2634977 |
| 18 | A/Kamigoto/1498/2013 | EPI_ISL_18001746 | EPI2634962 | EPI2634963 | EPI2634964 | EPI2634965 | EPI2634966 | EPI2634967 | EPI2634968 | EPI2634969 |
| 19 | A/Kamigoto/1502/2013 | EPI_ISL_18001715 | EPI2634954 | EPI2634955 | EPI2634956 | EPI2634957 | EPI2634958 | EPI2634959 | EPI2634960 | EPI2634961 |
| 20 | A/Kamigoto/1512/2013 | EPI_ISL_18001714 | EPI2634946 | EPI2634947 | EPI2634948 | EPI2634949 | EPI2634950 | EPI2634951 | EPI2634952 | EPI2634953 |
| 21 | A/Kamigoto/1542/2013 | EPI_ISL_18001713 | EPI2634937 | EPI2634938 | EPI2634939 | EPI2634940 | EPI2634941 | EPI2634942 | EPI2634943 | EPI2634944 |
| 22 | A/Kamigoto/1544/2013 | EPI_ISL_18001707 | EPI2634922 | EPI2634923 | EPI2634924 | EPI2634925 | EPI2634926 | EPI2634927 | EPI2634928 | EPI2634929 |
| 23 | A/Kamigoto/1547/2013 | EPI_ISL_18001706 | EPI2634865 | EPI2634887 | EPI2634908 | EPI2634917 | EPI2634918 | EPI2634919 | EPI2634920 | EPI2634921 |
| 24 | A/Kamigoto/1553/2013 | EPI_ISL_18001705 | EPI2634844 | EPI2634845 | EPI2634846 | EPI2634847 | EPI2634848 | EPI2634849 | EPI2634850 | EPI2634851 |
| 25 | A/Kamigoto/1588/2013 | EPI_ISL_18001704 | EPI2634835 | EPI2634837 | EPI2634838 | EPI2634839 | EPI2634840 | EPI2634841 | EPI2634842 | EPI2634843 |
| 26 | A/Kamigoto/1592/2013 | EPI_ISL_18001703 | EPI2634778 | EPI2634798 | EPI2634819 | EPI2634830 | EPI2634831 | EPI2634832 | EPI2634833 | EPI2634834 |
| 27 | A/Kamigoto/1596/2013 | EPI_ISL_18001702 | EPI2634504 | EPI2634523 | EPI2634543 | EPI2634563 | EPI2634578 | EPI2634600 | EPI2634618 | EPI2634641 |
| 28 | A/Kamigoto/1598/2013 | EPI_ISL_18001701 | EPI2634425 | EPI2634426 | EPI2634427 | EPI2634428 | EPI2634429 | EPI2634430 | EPI2634431 | EPI2634432 |
| 29 | A/Kamigoto/1601/2013 | EPI_ISL_18001686 | EPI2634336 | EPI2634350 | EPI2634362 | EPI2634374 | EPI2634387 | EPI2634401 | EPI2634412 | EPI2634415 |
| 30 | A/Kamigoto/1603/2013 | EPI_ISL_18001652 | EPI2634085 | EPI2634099 | EPI2634110 | EPI2634124 | EPI2634134 | EPI2634146 | EPI2634156 | EPI2634174 |
| 31 | A/Kamigoto/1607/2013 | EPI_ISL_18001626 | EPI2633893 | EPI2633905 | EPI2633917 | EPI2633935 | EPI2633946 | EPI2633958 | EPI2633971 | EPI2633981 |
| 32 | A/Kamigoto/1610/2013 | EPI_ISL_18001610 | EPI2633719 | EPI2633745 | EPI2633763 | EPI2633764 | EPI2633765 | EPI2633766 | EPI2633767 | EPI2633775 |
| 33 | A/Kamigoto/1778/2013 | EPI_ISL_18001609 | EPI2633703 | EPI2633704 | EPI2633705 | EPI2633706 | EPI2633707 | EPI2633708 | EPI2633709 | EPI2633710 |
| 34 | A/Kamigoto/1803/2013 | EPI_ISL_18001608 | EPI2633695 | EPI2633696 | EPI2633697 | EPI2633698 | EPI2633699 | EPI2633700 | EPI2633701 | EPI2633702 |
| 35 | A/Kamigoto/1804/2013 | EPI_ISL_18001607 | EPI2633687 | EPI2633688 | EPI2633689 | EPI2633690 | EPI2633691 | EPI2633692 | EPI2633693 | EPI2633694 |
| 36 | A/Kamigoto/1816/2013 | EPI_ISL_18001606 | EPI2633679 | EPI2633680 | EPI2633681 | EPI2633682 | EPI2633683 | EPI2633684 | EPI2633685 | EPI2633686 |
| 37 | A/Kamigoto/1821/2013 | EPI_ISL_18001522 | EPI2633671 | EPI2633672 | EPI2633673 | EPI2633674 | EPI2633675 | EPI2633676 | EPI2633677 | EPI2633678 |
| 38 | A/Kamigoto/1849/2013 | EPI_ISL_18001521 | EPI2633663 | EPI2633664 | EPI2633665 | EPI2633666 | EPI2633667 | EPI2633668 | EPI2633669 | EPI2633670 |
| 39 | A/Kamigoto/1856/2013 | EPI_ISL_18001520 | EPI2633655 | EPI2633656 | EPI2633657 | EPI2633658 | EPI2633659 | EPI2633660 | EPI2633661 | EPI2633662 |
| 40 | A/Kamigoto/1877/2013 | EPI_ISL_18001518 | EPI2633641 | EPI2633642 | EPI2633643 | EPI2633644 | EPI2633645 | EPI2633646 | EPI2633647 | EPI2633648 |
| 41 | A/Kamigoto/1881/2013 | EPI_ISL_18001517 | EPI2633633 | EPI2633634 | EPI2633635 | EPI2633636 | EPI2633637 | EPI2633638 | EPI2633639 | EPI2633640 |
| 42 | A/Kamigoto/1884/2013 | EPI_ISL_18001516 | EPI2633625 | EPI2633626 | EPI2633627 | EPI2633628 | EPI2633629 | EPI2633630 | EPI2633631 | EPI2633632 |
| 43 | A/Kamigoto/1896/2013 | EPI_ISL_18001514 | EPI2633617 | EPI2633618 | EPI2633619 | EPI2633620 | EPI2633621 | EPI2633622 | EPI2633623 | EPI2633624 |
| 44 | A/Kamigoto/1903/2013 | EPI_ISL_18001513 | EPI2633301 | EPI2633302 | EPI2633303 | EPI2633304 | EPI2633305 | EPI2633306 | EPI2633307 | EPI2633311 |
| 45 | A/Kamigoto/1913/2013 | EPI_ISL_18001512 | EPI2633293 | EPI2633294 | EPI2633295 | EPI2633296 | EPI2633297 | EPI2633298 | EPI2633299 | EPI2633300 |
| 46 | A/Kamigoto/1938/2013 | EPI_ISL_18001511 | EPI2633285 | EPI2633286 | EPI2633287 | EPI2633288 | EPI2633289 | EPI2633290 | EPI2633291 | EPI2633292 |
| 47 | A/Kamigoto/2014/2013 | EPI_ISL_18001510 | EPI2633277 | EPI2633278 | EPI2633279 | EPI2633280 | EPI2633281 | EPI2633282 | EPI2633283 | EPI2633284 |
| 48 | A/Kamigoto/1409/2013 | EPI_ISL_17785996 | EPI2589356 | EPI2589357 | EPI2589358 | EPI2589359 | EPI2589360 | EPI2589361 | EPI2589362 | EPI2589363 |
| 49 | A/Kamigoto/1407/2013 | EPI_ISL_17785995 | EPI2589348 | EPI2589349 | EPI2589350 | EPI2589351 | EPI2589352 | EPI2589353 | EPI2589354 | EPI2589355 |
| 50 | A/Kamigoto/1399/2013 | EPI_ISL_17785743 | EPI2589340 | EPI3785004 | EPI3785005 | EPI2589343 | EPI2589344 | EPI2589345 | EPI2589346 | EPI2589347 |
| 51 | A/Kamigoto/1397/2013 | EPI_ISL_17785739 | EPI2589324 | EPI2589325 | EPI3785006 | EPI2589327 | EPI2589328 | EPI2589329 | EPI2589330 | EPI2589331 |
| 52 | A/Kamigoto/1396/2013 | EPI_ISL_17785738 | EPI2589316 | EPI2589317 | EPI2589318 | EPI2589319 | EPI2589320 | EPI2589321 | EPI2589322 | EPI2589323 |
| 53 | A/Kamigoto/1389/2013 | EPI_ISL_17785724 | EPI2589308 | EPI2589309 | EPI2589310 | EPI2589311 | EPI2589312 | EPI2589313 | EPI2589314 | EPI2589315 |
| 54 | A/Kamigoto/1386/2013 | EPI_ISL_17785723 | EPI2589228 | EPI2589229 | EPI2589230 | EPI2589231 | EPI2589232 | EPI2589233 | EPI2589234 | EPI2589235 |
| 55 | A/Kamigoto/1385/2013 | EPI_ISL_17785722 | EPI2589220 | EPI2589221 | EPI2589222 | EPI2589223 | EPI2589224 | EPI2589225 | EPI2589226 | EPI2589227 |
| 56 | A/Kamigoto/1382/2013 | EPI_ISL_17785719 | EPI2589212 | EPI2589213 | EPI2589214 | EPI2589215 | EPI2589216 | EPI2589217 | EPI2589218 | EPI2589219 |
| 57 | A/Kamigoto/1381/2013 | EPI_ISL_17785716 | EPI2589204 | EPI2589205 | EPI2589206 | EPI2589207 | EPI2589208 | EPI2589209 | EPI2589210 | EPI2589211 |
| 58 | A/Kamigoto/1378/2013 | EPI_ISL_17785715 | EPI2589196 | EPI2589197 | EPI2589198 | EPI2589199 | EPI2589200 | EPI2589201 | EPI2589202 | EPI2589203 |
| 59 | A/Kamigoto/1376/2013 | EPI_ISL_17785627 | EPI2588519 | EPI3785007 | EPI2588521 | EPI2588522 | EPI2588523 | EPI2588524 | EPI3785008 | EPI2588525 |
| 60 | A/Kamigoto/1375/2013 | EPI_ISL_17785624 | EPI2588511 | EPI2588512 | EPI2588513 | EPI2588514 | EPI2588515 | EPI2588516 | EPI2588517 | EPI2588518 |
| 61 | A/Kamigoto/1373/2013 | EPI_ISL_17785619 | EPI2588503 | EPI2588504 | EPI2588505 | EPI2588506 | EPI2588507 | EPI2588508 | EPI2588509 | EPI2588510 |
| 62 | A/Kamigoto/1370/2013 | EPI_ISL_17785617 | EPI2588495 | EPI2588496 | EPI2588497 | EPI2588498 | EPI2588499 | EPI2588500 | EPI2588501 | EPI2588502 |
| 63 | A/Kamigoto/1369/2013 | EPI_ISL_17785616 | EPI2588487 | EPI2588488 | EPI2588489 | EPI2588490 | EPI2588491 | EPI2588492 | EPI2588493 | EPI2588494 |
| 64 | A/Kamigoto/1368/2013 | EPI_ISL_17785615 | EPI2588479 | EPI2588480 | EPI2588481 | EPI2588482 | EPI2588483 | EPI2588484 | EPI2588485 | EPI2588486 |
| 65 | A/Kamigoto/1367/2013 | EPI_ISL_17785614 | EPI2588471 | EPI2588472 | EPI2588473 | EPI2588474 | EPI2588475 | EPI2588476 | EPI2588477 | EPI2588478 |
| 66 | A/Kamigoto/1317/2013 | EPI_ISL_17761516 | EPI2582626 | EPI2582627 | EPI2582628 | EPI2582629 | EPI2582630 | EPI2582631 | EPI2582632 | EPI2582633 |
| 67 | A/Kamigoto/1301/2013 | EPI_ISL_17739156 | EPI2580996 | EPI2580997 | EPI2580998 | EPI2580999 | EPI2581000 | EPI2581001 | EPI2581002 | EPI2581003 |
| 68 | A/Kamigoto/1262/2013 | EPI_ISL_17738971 | EPI2580988 | EPI2580989 | EPI3785017 | EPI2580991 | EPI2580992 | EPI2580993 | EPI2580994 | EPI2580995 |
| 69 | A/Kamigoto/1248/2013 | EPI_ISL_17738944 | EPI2580980 | EPI2580981 | EPI2580982 | EPI2580983 | EPI2580984 | EPI2580985 | EPI2580986 | EPI2580987 |
| 70 | A/Kamigoto/1244/2013 | EPI_ISL_17730092 | EPI2579582 | EPI2579583 | EPI2579584 | EPI2579585 | EPI2579586 | EPI2579587 | EPI2579588 | EPI2579589 |
| 71 | A/Kamigoto/1240/2013 | EPI_ISL_17730088 | EPI2579574 | EPI2579575 | EPI2579576 | EPI2579577 | EPI2579578 | EPI2579579 | EPI2579580 | EPI2579581 |
| 72 | A/Kamigoto/1239/2013 | EPI_ISL_17730086 | EPI2579566 | EPI2579567 | EPI2579568 | EPI2579569 | EPI2579570 | EPI2579571 | EPI2579572 | EPI2579573 |
| 73 | A/Kamigoto/1236/2013 | EPI_ISL_17730085 | EPI2579558 | EPI2579559 | EPI2579560 | EPI2579561 | EPI2579562 | EPI2579563 | EPI2579564 | EPI2579565 |
| 74 | A/Kamigoto/1215/2013 | EPI_ISL_17730082 | EPI2579542 | EPI2579543 | EPI2579544 | EPI2579545 | EPI2579546 | EPI2579547 | EPI2579548 | EPI2579549 |
| 75 | A/Kamigoto/1210/2013 | EPI_ISL_17730079 | EPI2579534 | EPI2579535 | EPI2579536 | EPI2579537 | EPI2579538 | EPI2579539 | EPI2579540 | EPI2579541 |
| 76 | A/Kamigoto/1182/2013 | EPI_ISL_17730075 | EPI2579526 | EPI2579527 | EPI2579528 | EPI2579529 | EPI2579530 | EPI2579531 | EPI2579532 | EPI2579533 |
| 77 | A/Kamigoto/1180/2013 | EPI_ISL_17730072 | EPI2579517 | EPI2579518 | EPI2579519 | EPI2579520 | EPI2579521 | EPI2579523 | EPI2579524 | EPI2579525 |
| 78 | A/Kamigoto/1177/2013 | EPI_ISL_17730069 | EPI2579508 | EPI2579509 | EPI2579511 | EPI2579512 | EPI2579513 | EPI2579514 | EPI2579515 | EPI2579516 |
| 79 | A/Kamigoto/1172/2013 | EPI_ISL_17730068 | EPI2579500 | EPI2579501 | EPI2579502 | EPI2579503 | EPI2579504 | EPI2579505 | EPI2579506 | EPI2579507 |
| 80 | A/Kamigoto/1169/2013 | EPI_ISL_17730067 | EPI2579492 | EPI2579493 | EPI2579494 | EPI2579495 | EPI2579496 | EPI2579497 | EPI2579498 | EPI2579499 |
| 81 | A/Kamigoto/1164/2013 | EPI_ISL_17730066 | EPI2579484 | EPI2579485 | EPI2579486 | EPI2579487 | EPI2579488 | EPI2579489 | EPI2579490 | EPI2579491 |
| 82 | A/Kamigoto/1162/2013 | EPI_ISL_17730065 | EPI2579476 | EPI2579477 | EPI2579478 | EPI2579479 | EPI2579480 | EPI2579481 | EPI2579482 | EPI2579483 |
| 83 | A/Kamigoto/1161/2013 | EPI_ISL_17730047 | EPI2579468 | EPI2579469 | EPI2579470 | EPI2579471 | EPI2579472 | EPI2579473 | EPI2579474 | EPI2579475 |
| 84 | A/Kamigoto/1156/2013 | EPI_ISL_17726035 | EPI2579164 | EPI2579165 | EPI2579166 | EPI2579167 | EPI2579168 | EPI2579169 | EPI2579170 | EPI2579171 |
| 85 | A/Kamigoto/1155/2013 | EPI_ISL_17692316 | EPI2563277 | EPI2563279 | EPI2563278 | EPI2579159 | EPI2579160 | EPI2579161 | EPI2579162 | EPI2579163 |
| 86 | A/Kamigoto/1154/2013 | EPI_ISL_17692307 | EPI2563266 | EPI2563269 | EPI2563268 | EPI2563270 | EPI2563271 | EPI2563272 | EPI2563273 | EPI2563274 |
| 87 | A/Kamigoto/1149/2013 | EPI_ISL_17692279 | EPI2563258 | EPI2563259 | EPI2563260 | EPI2563261 | EPI2563262 | EPI2563263 | EPI2563264 | EPI2563265 |
| 88 | A/Kamigoto/1144/2013 | EPI_ISL_17692278 | EPI2563250 | EPI2563251 | EPI2563252 | EPI2563253 | EPI2563254 | EPI2563255 | EPI2563256 | EPI2563257 |
| 89 | A/Kamigoto/1132/2013 | EPI_ISL_17692276 | EPI2563242 | EPI2563243 | EPI2563244 | EPI2563245 | EPI2563246 | EPI2563247 | EPI2563248 | EPI2563249 |
| 90 | A/Kamigoto/1122/2013 | EPI_ISL_17692275 | EPI2563234 | EPI2563235 | EPI2563236 | EPI2563237 | EPI2563238 | EPI2563239 | EPI2563240 | EPI2563241 |
| 91 | A/Kamigoto/1106/2013 | EPI_ISL_17692272 | EPI2563222 | EPI2563223 | EPI2563224 | EPI2563225 | EPI2563226 | EPI2563227 | EPI2563228 | EPI2563229 |
| 92 | A/Kamigoto/1097/2013 | EPI_ISL_17692271 | EPI2579550 | EPI2579551 | EPI2579552 | EPI2579553 | EPI2579554 | EPI2579555 | EPI2579556 | EPI2579557 |
| 93 | A/Kamigoto/1095/2013 | EPI_ISL_17692267 | EPI2563214 | EPI3785009 | EPI2563216 | EPI2563217 | EPI2563218 | EPI2563219 | EPI2563220 | EPI2563221 |
| 94 | A/Kamigoto/1092/2013 | EPI_ISL_17692266 | EPI2563206 | EPI2563207 | EPI2563208 | EPI2563209 | EPI2563210 | EPI2563211 | EPI2563212 | EPI2563213 |
| 95 | A/Kamigoto/1081/2013 | EPI_ISL_17692265 | EPI2563198 | EPI2563199 | EPI2563200 | EPI2563201 | EPI2563202 | EPI2563203 | EPI2563204 | EPI2563205 |
| 96 | A/Kamigoto/1068/2013 | EPI_ISL_17692264 | EPI2563190 | EPI2563191 | EPI2563192 | EPI2563193 | EPI2563194 | EPI2563195 | EPI2563196 | EPI2563197 |
| 97 | A/Kamigoto/1067/2013 | EPI_ISL_17692257 | EPI2563182 | EPI2563183 | EPI2563184 | EPI2563185 | EPI2563186 | EPI2563187 | EPI2563188 | EPI2563189 |
| 98 | A/Kamigoto/1064/2013 | EPI_ISL_17692212 | EPI2563174 | EPI2563175 | EPI2563176 | EPI2563177 | EPI2563178 | EPI2563179 | EPI2563180 | EPI2563181 |
| 99 | A/Kamigoto/1060/2013 | EPI_ISL_17692112 | EPI2563156 | EPI2563157 | EPI2563158 | EPI2563159 | EPI2563160 | EPI2563161 | EPI2563162 | EPI2563163 |
| 100 | A/Kamigoto/1052/2013 | EPI_ISL_17692053 | EPI2563148 | EPI2563149 | EPI2563150 | EPI2563151 | EPI2563152 | EPI2563153 | EPI2563154 | EPI2563155 |
| 101 | A/Kamigoto/1050/2013 | EPI_ISL_17692052 | EPI2563140 | EPI2563141 | EPI2563142 | EPI2563143 | EPI2563144 | EPI2563145 | EPI2563146 | EPI2563147 |
| 102 | A/Kamigoto/1038/2013 | EPI_ISL_17692051 | EPI2563132 | EPI2563133 | EPI2563134 | EPI2563135 | EPI2563136 | EPI2563137 | EPI2563138 | EPI2563139 |
| 103 | A/Kamigoto/1036/2013 | EPI_ISL_17692004 | EPI2563032 | EPI2563033 | EPI2563034 | EPI2563035 | EPI2563036 | EPI2563037 | EPI2563038 | EPI2563039 |
| 104 | A/Kamigoto/1035/2013 | EPI_ISL_17691998 | EPI2563024 | EPI2563025 | EPI2563026 | EPI2563027 | EPI2563028 | EPI2563029 | EPI2563030 | EPI2563031 |
| 105 | A/Kamigoto/1026/2013 | EPI_ISL_17691980 | EPI2563016 | EPI2563017 | EPI2563018 | EPI2563019 | EPI2563020 | EPI2563021 | EPI2563022 | EPI2563023 |
| 106 | A/Kamigoto/1020/2013 | EPI_ISL_17691968 | EPI2563001 | EPI2563004 | EPI2563006 | EPI2563008 | EPI2563009 | EPI2563011 | EPI2563012 | EPI2563014 |
| 107 | A/Kamigoto/1006/2013 | EPI_ISL_17691966 | EPI2562992 | EPI2562993 | EPI2562994 | EPI2562995 | EPI2562996 | EPI2562997 | EPI2562998 | EPI2562999 |
| 108 | A/Kamigoto/994/2013 | EPI_ISL_17691965 | EPI2562984 | EPI2562985 | EPI2562986 | EPI2562987 | EPI2562988 | EPI2562989 | EPI2562990 | EPI2562991 |
| 109 | A/Kamigoto/992/2013 | EPI_ISL_17689263 | EPI2562656 | EPI2562657 | EPI2562658 | EPI2562659 | EPI2562660 | EPI2562661 | EPI2562662 | EPI2562663 |
| 110 | A/Kamigoto/920/2013 | EPI_ISL_17684817 | EPI2562648 | EPI2562649 | EPI2562650 | EPI2562651 | EPI2562652 | EPI2562653 | EPI2562654 | EPI2562655 |
| 111 | A/Kamigoto/878/2013 | EPI_ISL_17684804 | EPI2562640 | EPI2562641 | EPI2562642 | EPI2562643 | EPI2562644 | EPI2562645 | EPI2562646 | EPI2562647 |
| 112 | A/Kamigoto/722/2013 | EPI_ISL_17684786 | EPI2562622 | EPI2562623 | EPI2562624 | EPI2562625 | EPI2562626 | EPI2562627 | EPI2562628 | EPI2562629 |
| 113 | A/Kamigoto/707/2013 | EPI_ISL_17684785 | EPI2562606 | EPI2562607 | EPI2562608 | EPI2562609 | EPI2562610 | EPI2562612 | EPI2562614 | EPI2562616 |
| 114 | A/Kamigoto/667/2013 | EPI_ISL_17684676 | EPI2562597 | EPI2562598 | EPI2562599 | EPI2562600 | EPI2562601 | EPI2562602 | EPI2562603 | EPI2562604 |
| 115 | A/Kamigoto/666/2013 | EPI_ISL_17684609 | EPI2562584 | EPI2562585 | EPI2562587 | EPI2562588 | EPI2562589 | EPI2562590 | EPI2562591 | EPI2562593 |
| 116 | A/Kamigoto/665/2013 | EPI_ISL_17684607 | EPI2562575 | EPI2562576 | EPI2562578 | EPI2562579 | EPI2562580 | EPI2562581 | EPI2562582 | EPI2562583 |
| 117 | A/Kamigoto/635/2013 | EPI_ISL_17684606 | EPI2562567 | EPI2562568 | EPI2562569 | EPI2562570 | EPI2562571 | EPI2562572 | EPI2562573 | EPI2562574 |
| 118 | A/Kamigoto/624/2013 | EPI_ISL_17684520 | EPI2562559 | EPI2562560 | EPI2562561 | EPI2562562 | EPI2562563 | EPI2562564 | EPI2562565 | EPI2562566 |
| 119 | A/Kamigoto/618/2013 | EPI_ISL_17684519 | EPI2562551 | EPI2562552 | EPI2562553 | EPI2562554 | EPI2562555 | EPI2562556 | EPI2562557 | EPI2562558 |
| 120 | A/Kamigoto/617/2013 | EPI_ISL_17684518 | EPI2562543 | EPI2562544 | EPI2562545 | EPI2562546 | EPI2562547 | EPI2562548 | EPI2562549 | EPI2562550 |
| 121 | A/Kamigoto/616/2013 | EPI_ISL_17683953 | EPI2562535 | EPI2562536 | EPI2562537 | EPI2562538 | EPI2562539 | EPI2562540 | EPI2562541 | EPI2562542 |
| 122 | A/Kamigoto/615/2013 | EPI_ISL_17683857 | EPI2562527 | EPI2562528 | EPI2562529 | EPI2562530 | EPI2562531 | EPI2562532 | EPI2562533 | EPI2562534 |
| 123 | A/Kamigoto/614/2013 | EPI_ISL_17683792 | EPI2562519 | EPI2562520 | EPI2562521 | EPI2562522 | EPI2562523 | EPI2562524 | EPI2562525 | EPI2562526 |
| 124 | A/Kamigoto/612/2013 | EPI_ISL_17683791 | EPI2562511 | EPI2562512 | EPI2562513 | EPI2562514 | EPI2562515 | EPI2562516 | EPI2562517 | EPI2562518 |
| 125 | A/Kamigoto/603/2013 | EPI_ISL_17683790 | EPI2562503 | EPI2562504 | EPI2562505 | EPI2562506 | EPI2562507 | EPI2562508 | EPI2562509 | EPI2562510 |
| 126 | A/Kamigoto/600/2013 | EPI_ISL_17683728 | EPI2562495 | EPI2562496 | EPI2562497 | EPI2562498 | EPI2562499 | EPI2562500 | EPI2562501 | EPI2562502 |
| 127 | A/Kamigoto/590/2013 | EPI_ISL_17683727 | EPI2562487 | EPI2562488 | EPI2562489 | EPI2562490 | EPI2562491 | EPI2562492 | EPI2562493 | EPI2562494 |
| 128 | A/Kamigoto/561/2013 | EPI_ISL_17683726 | EPI3785010 | EPI2562480 | EPI2562481 | EPI2562482 | EPI2562483 | EPI2562484 | EPI2562485 | EPI2562486 |
| 129 | A/Kamigoto/480/2013 | EPI_ISL_17683724 | EPI2562463 | EPI2562464 | EPI2562465 | EPI2562466 | EPI2562467 | EPI2562468 | EPI2562469 | EPI2562470 |
| 130 | A/Kamigoto/475/2013 | EPI_ISL_17683723 | EPI2562455 | EPI2562456 | EPI2562457 | EPI2562458 | EPI2562459 | EPI2562460 | EPI2562461 | EPI2562462 |
| 131 | A/Kamigoto/375/2013 | EPI_ISL_17683722 | EPI2562447 | EPI2562448 | EPI2562449 | EPI2562450 | EPI2562451 | EPI2562452 | EPI2562453 | EPI2562454 |
| 132 | A/Kamigoto/310/2013 | EPI_ISL_17683293 | EPI2562434 | EPI2562435 | EPI2562436 | EPI2562437 | EPI2562438 | EPI2562439 | EPI2562440 | EPI2562441 |
| 133 | A/Kamigoto/274/2013 | EPI_ISL_17683291 | EPI2562420 | EPI2562421 | EPI2562422 | EPI2562423 | EPI2562424 | EPI2562425 | EPI2562426 | EPI2562427 |
| 134 | A/Kamigoto/273/2013 | EPI_ISL_17683048 | EPI2562412 | EPI2562413 | EPI2562414 | EPI2562415 | EPI2562416 | EPI2562417 | EPI2562418 | EPI2562419 |
| 135 | A/Kamigoto/220/2013 | EPI_ISL_17683047 | EPI2562404 | EPI2562405 | EPI2562406 | EPI2562407 | EPI2562408 | EPI2562409 | EPI2562410 | EPI2562411 |
| 136 | A/Kamigoto/216/2013 | EPI_ISL_17683046 | EPI2562396 | EPI2562397 | EPI2562398 | EPI2562399 | EPI2562400 | EPI2562401 | EPI2562402 | EPI2562403 |
| 137 | A/Kamigoto/208/2013 | EPI_ISL_17683045 | EPI2562388 | EPI2562389 | EPI2562390 | EPI2562391 | EPI2562392 | EPI2562393 | EPI2562394 | EPI2562395 |
| 138 | A/Kamigoto/1137/2012 | EPI_ISL_17296154 | EPI2477423 | EPI2477424 | EPI2477425 | EPI2477426 | EPI2477427 | EPI2477428 | EPI2477429 | EPI2477430 |
| 139 | A/Kamigoto/1209/2012 | EPI_ISL_17296116 | EPI2477415 | EPI2477416 | EPI2477417 | EPI2477418 | EPI2477419 | EPI2477420 | EPI2477421 | EPI2477422 |
| 140 | A/Kamigoto/1378/2012 | EPI_ISL_17296115 | EPI2477407 | EPI2477408 | EPI2477409 | EPI2477410 | EPI2477411 | EPI2477412 | EPI2477413 | EPI2477414 |
| 141 | A/Kamigoto/1430/2012 | EPI_ISL_17296114 | EPI2477399 | EPI2477400 | EPI2477401 | EPI2477402 | EPI2477403 | EPI2477404 | EPI2477405 | EPI2477406 |
| 142 | A/Kamigoto/1437/2012 | EPI_ISL_17296113 | EPI2477391 | EPI2477392 | EPI2477393 | EPI2477394 | EPI2477395 | EPI2477396 | EPI2477397 | EPI2477398 |
| 143 | A/Kamigoto/1439/2012 | EPI_ISL_17296112 | EPI2477383 | EPI2477384 | EPI2477385 | EPI2477386 | EPI2477387 | EPI2477388 | EPI2477389 | EPI2477390 |
| 144 | A/Kamigoto/1483/2012 | EPI_ISL_17296108 | EPI2477375 | EPI2477376 | EPI2477377 | EPI2477378 | EPI2477379 | EPI2477380 | EPI2477381 | EPI2477382 |
| 145 | A/Kamigoto/1490/2012 | EPI_ISL_17296105 | EPI2477367 | EPI2477368 | EPI2477369 | EPI2477370 | EPI2477371 | EPI2477372 | EPI2477373 | EPI2477374 |
| 146 | A/Kamigoto/1514/2012 | EPI_ISL_17296097 | EPI2477359 | EPI2477360 | EPI2477361 | EPI2477362 | EPI2477363 | EPI2477364 | EPI2477365 | EPI2477366 |
| 147 | A/Kamigoto/1530/2012 | EPI_ISL_17296096 | EPI2477351 | EPI3785011 | EPI2477353 | EPI2477354 | EPI2477355 | EPI2477356 | EPI2477357 | EPI2477358 |
| 148 | A/Kamigoto/894/2012 | EPI_ISL_17296073 | EPI2477343 | EPI2477344 | EPI2477345 | EPI2477346 | EPI2477347 | EPI2477348 | EPI2477349 | EPI2477350 |
| 149 | A/Kamigoto/887/2012 | EPI_ISL_17296061 | EPI2477335 | EPI2477336 | EPI2477337 | EPI2477338 | EPI2477339 | EPI2477340 | EPI2477341 | EPI2477342 |
| 150 | A/Kamigoto/886/2012 | EPI_ISL_17296025 | EPI2477327 | EPI3785012 | EPI2477329 | EPI2477330 | EPI2477331 | EPI2477332 | EPI2477333 | EPI2477334 |
| 151 | A/Kamigoto/871/2012 | EPI_ISL_17295949 | EPI3785013 | EPI2477320 | EPI2477321 | EPI2477322 | EPI2477323 | EPI2477324 | EPI2477325 | EPI2477326 |
| 152 | A/Kamigoto/805/2012 | EPI_ISL_17295903 | EPI3785014 | EPI2477312 | EPI2477313 | EPI2477314 | EPI2477315 | EPI2477316 | EPI2477317 | EPI2477318 |
| 153 | A/Kamigoto/796/2012 | EPI_ISL_17295902 | EPI2477303 | EPI3785015 | EPI2477305 | EPI2477306 | EPI2477307 | EPI2477308 | EPI2477309 | EPI2477310 |
| 154 | A/Kamigoto/784/2012 | EPI_ISL_17295671 | EPI2477295 | EPI2477296 | EPI2477297 | EPI2477298 | EPI2477299 | EPI2477300 | EPI2477301 | EPI2477302 |
| 155 | A/Kamigoto/772/2012 | EPI_ISL_17104742 | EPI2451325 | EPI2451326 | EPI2451327 | EPI2451328 | EPI2451329 | EPI2451330 | EPI2451331 | EPI2451332 |
| 156 | A/Kamigoto/771/2012 | EPI_ISL_17104741 | EPI2451317 | EPI2451318 | EPI2451319 | EPI2451320 | EPI2451321 | EPI2451322 | EPI2451323 | EPI2451324 |
| 157 | A/Kamigoto/765/2012 | EPI_ISL_17104740 | EPI2451309 | EPI2451310 | EPI2451311 | EPI2451312 | EPI2451313 | EPI2451314 | EPI2451315 | EPI2451316 |
| 158 | A/Kamigoto/763/2012 | EPI_ISL_17104739 | EPI2451301 | EPI2451302 | EPI2451303 | EPI2451304 | EPI2451305 | EPI2451306 | EPI2451307 | EPI2451308 |
| 159 | A/Kamigoto/762/2012 | EPI_ISL_17104738 | EPI2451293 | EPI2451294 | EPI2451295 | EPI2451296 | EPI2451297 | EPI2451298 | EPI2451299 | EPI2451300 |
| 160 | A/Kamigoto/757/2012 | EPI_ISL_17104737 | EPI2451285 | EPI2451286 | EPI2451287 | EPI2451288 | EPI2451289 | EPI2451290 | EPI2451291 | EPI2451292 |
| 161 | A/Kamigoto/752/2012 | EPI_ISL_17104735 | EPI2451277 | EPI2451278 | EPI2451279 | EPI2451280 | EPI2451281 | EPI2451282 | EPI2451283 | EPI2451284 |
| 162 | A/Kamigoto/1460/2012 | EPI_ISL_17103548 | EPI2449375 | EPI2449376 | EPI2449377 | EPI2449378 | EPI2449379 | EPI2449380 | EPI2449381 | EPI2449382 |
| 163 | A/Kamigoto/749/2012 | EPI_ISL_17103148 | EPI2449359 | EPI2449360 | EPI2449361 | EPI2449362 | EPI2449363 | EPI2449364 | EPI2449365 | EPI2449366 |
| 164 | A/Kamigoto/729/1012 | EPI_ISL_17103065 | EPI2449342 | EPI2449344 | EPI3785016 | EPI2449346 | EPI2449347 | EPI2449348 | EPI2449349 | EPI2449350 |
| 165 | A/Kamigoto/1075/2012 | EPI_ISL_17103062 | EPI2449319 | EPI2449320 | EPI2449321 | EPI2449322 | EPI2449323 | EPI2449324 | EPI2449325 | EPI2449326 |
| 166 | A/Kamigoto/175/2013 | EPI_ISL_17092239 | EPI2440003 | EPI2440052 | EPI2440085 | EPI2440098 | EPI2440099 | EPI2440100 | EPI2440101 | EPI2440102 |
| 167 | A/Kamigoto/296/2013 | EPI_ISL_17683292 |  |  | EPI2562428 | EPI2562429 | EPI2562430 | EPI2562431 | EPI2562432 | EPI2562433 |
| 168 | A/Kamigoto/805/2013 | EPI_ISL_17684787 |  |  |  | EPI2562630 | EPI2562631 | EPI2562632 | EPI2562633 | EPI2562634 |
| 169 | A/Kamigoto/817/2013 | EPI_ISL_17684803 |  |  |  | EPI2562635 | EPI2562636 | EPI2562637 | EPI2562638 | EPI2562639 |
| 170 | A/Kamigoto/1247/2013 | EPI_ISL_17730095 |  |  |  | EPI2579590 | EPI2579591 | EPI2579592 | EPI2579593 | EPI2579594 |
| 171 | A/Kamigoto/1356/2013 | EPI_ISL_17761517 |  |  |  | EPI2582636 | EPI2582637 | EPI2582634 | EPI2582639 | EPI2582640 |
| 172 | A/Kamigoto/1360/2013 | EPI_ISL_1785613 |  |  | EPI2588465 | EPI2588466 | EPI2588467 | EPI2588468 | EPI2588469 | EPI2588470 |
| 173 | A/Kamigoto/2166/2013 | EPI_ISL_18000336 |  |  |  | EPI2632301 | EPI2632302 | EPI2632304 | EPI2632306 | EPI2632308 |
| 174 | A/Kamigoto/2164/2013 | EPI_ISL_18000338 |  | EPI2632314 | EPI2632315 | EPI2632316 | EPI2632317 | EPI2632318 | EPI2632319 | EPI2632320 |
| 175 | A/Kamigoto/2146/2013 | EPI_ISL_18000339 |  |  |  | EPI2632321 | EPI2632322 | EPI2632323 | EPI2632324 | EPI2632325 |
| 176 | A/Kamigoto/2134/2013 | EPI_ISL_18000486 |  |  |  | EPI2633272 | EPI2633273 | EPI2633274 | EPI2633275 | EPI2633276 |
| 177 | A/Kamigoto/1876/2013 | EPI_ISL_18001519 |  |  | EPI2633649 | EPI2633650 | EPI2633651 | EPI2633652 | EPI2633653 | EPI2633654 |
| 178 | A/Kamigoto/296/2013 | EPI_ISL_17683292 |  |  | EPI2562428 | EPI2562429 | EPI2562430 | EPI2562431 | EPI2562432 | EPI2562433 |

|  |  |  |  |  |  |  |  |  |  |
| --- | --- | --- | --- | --- | --- | --- | --- | --- | --- |
|  |  |  |  |  |  |  |  |  |  |
|  |  |  |  |  |  |  |  |  |  |
|  |  |  |  |  |  |  |  |  |  |
|  |  |  |  |  |  |  |  |  |  |
|  |  |  |  |  |  |  |  |  |  |
|  |  |  |  |  |  |  |  |  |  |
|  |  |  |  |  |  |  |  |  |  |
|  |  |  |  |  |  |  |  |  |  |
|  |  |  |  |  |  |  |  |  |  |
|  |  |  |  |  |  |  |  |  |  |
|  |  |  |  |  |  |  |  |  |  |


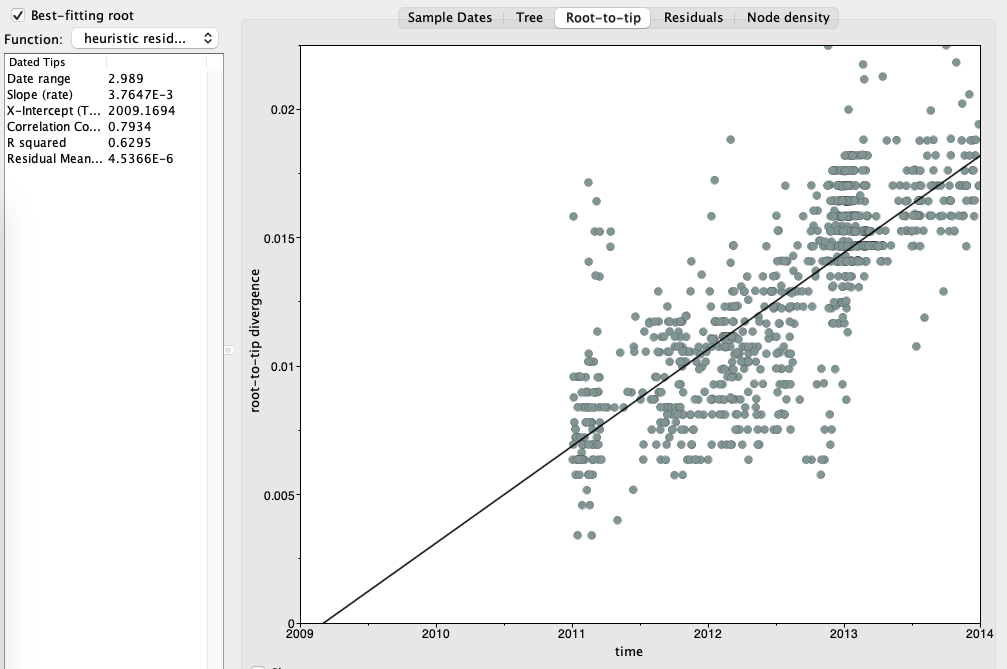


**FIGURE S-1.** Regression of root-to-tip genetic distances against sample collection dates.

**
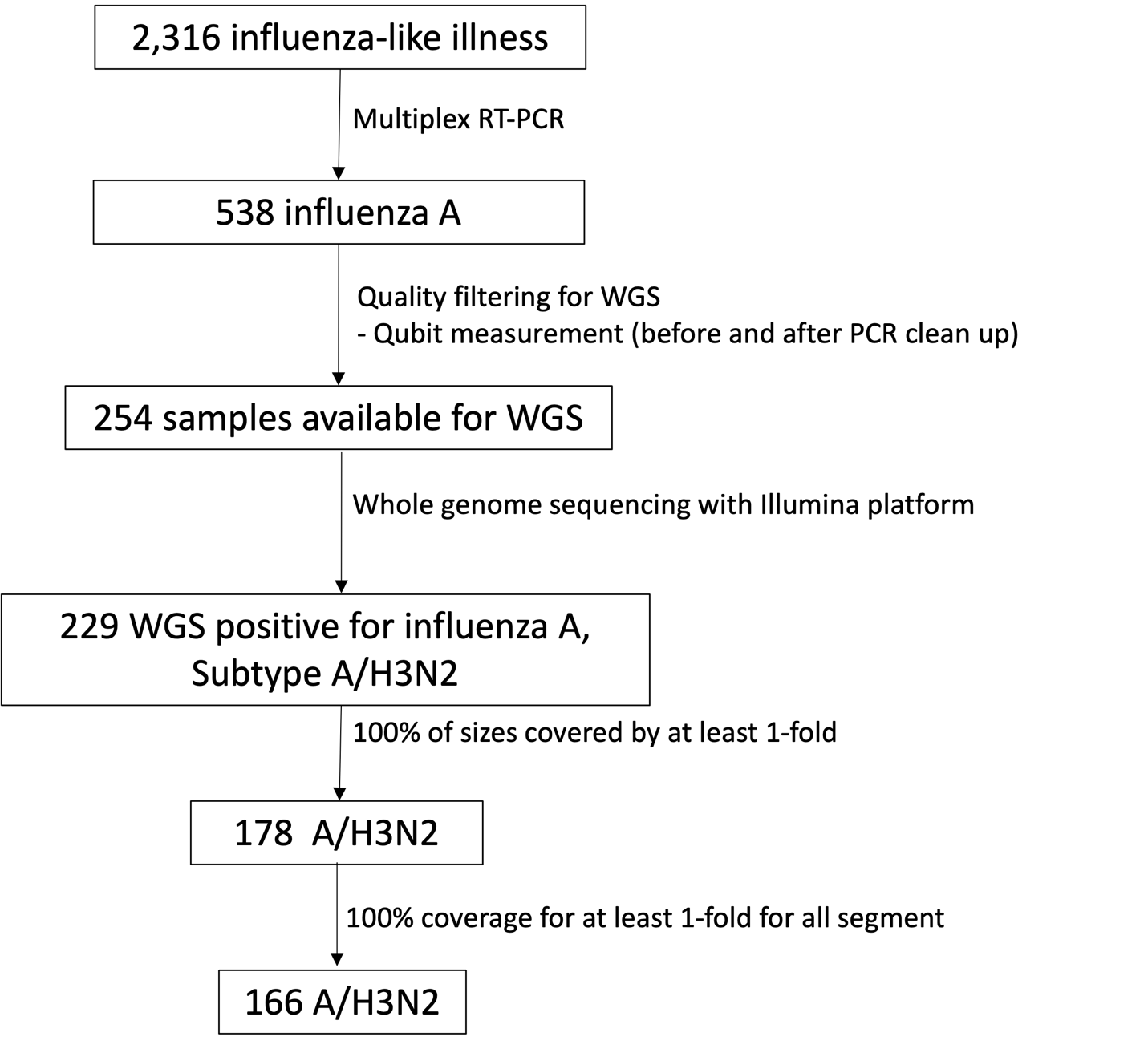
**

**FIGURE S-2:** Flowchart showing from samples collection to whole genome sequencing (WGS). 25 of the 254 samples available for WGS were discarded as subtype was not available


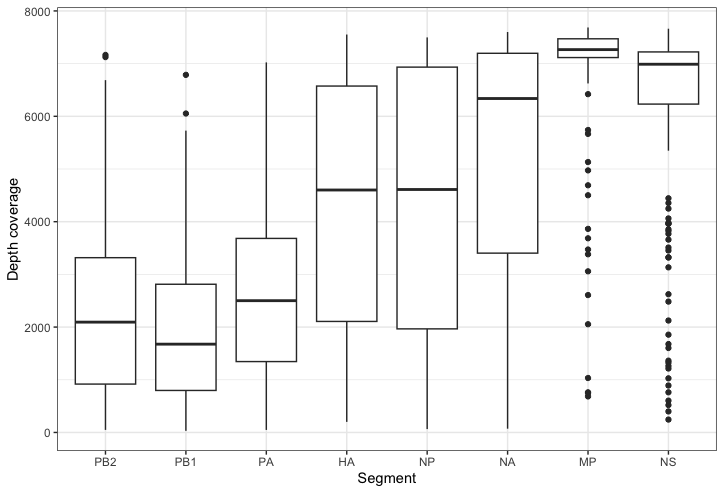


**FIGURE S-3:** Boxplots illustrating median read depth across each segment for A/H3N2 virus Boxes extend to the 1^st^ and 3^rd^ quartile.

The figure is in tiff file as separate document

**FIGURE S-4.** Time resolved phylogenetic tree of HA segments of Kamigoto island, Japan and global sequences (GISAID) as of 1^st^ December 2022 submission date.


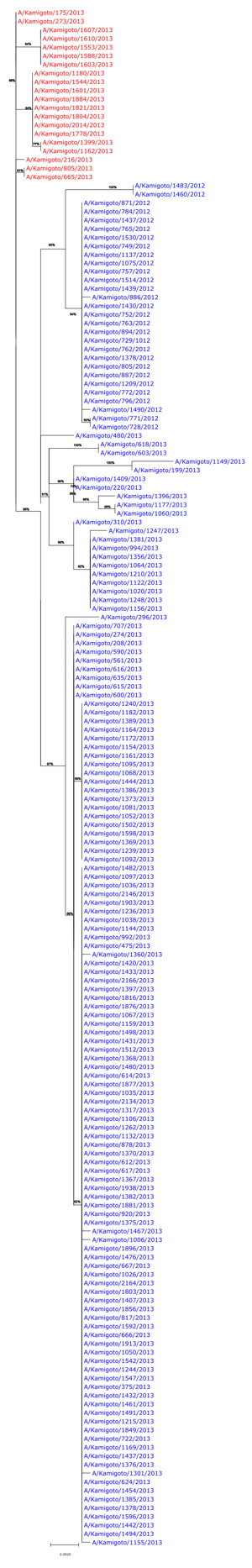


Clade 3C.2

Clade 3C.3a

Figure S-5: Maximum likelihood Phylogenetic tree of HA segments of A(H3N2) viruses from Kamigoto island, Japan . The sequences are colored by season (Red 2011/12 , Blue 2012/2023)


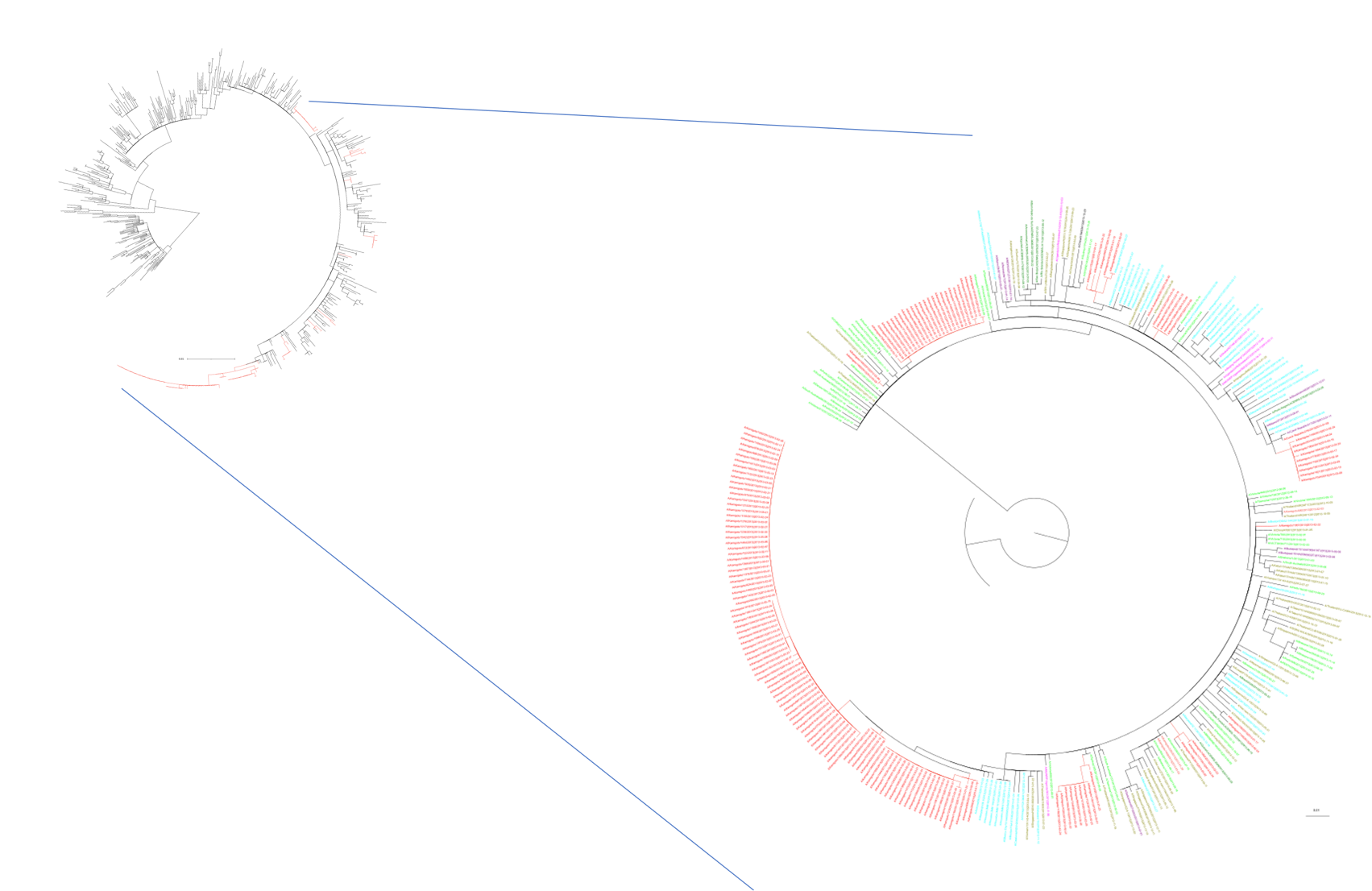


**Figure S-6:**  **Maximum-likelihood phylogenetic trees of PB2 segments of influenza A/H3N2 viruses circulating in Kamigoto and comparing sequences from strains isolated in Japan and other parts of the world from GISAID collected between 2011 and 2013.Kamigoto sequences are in red colour. The remaining strains are colored coded by region: North America in cyan, South America in teal, Oceania in green, Africa in magenta, Europe in purple, and Asia in brown.**


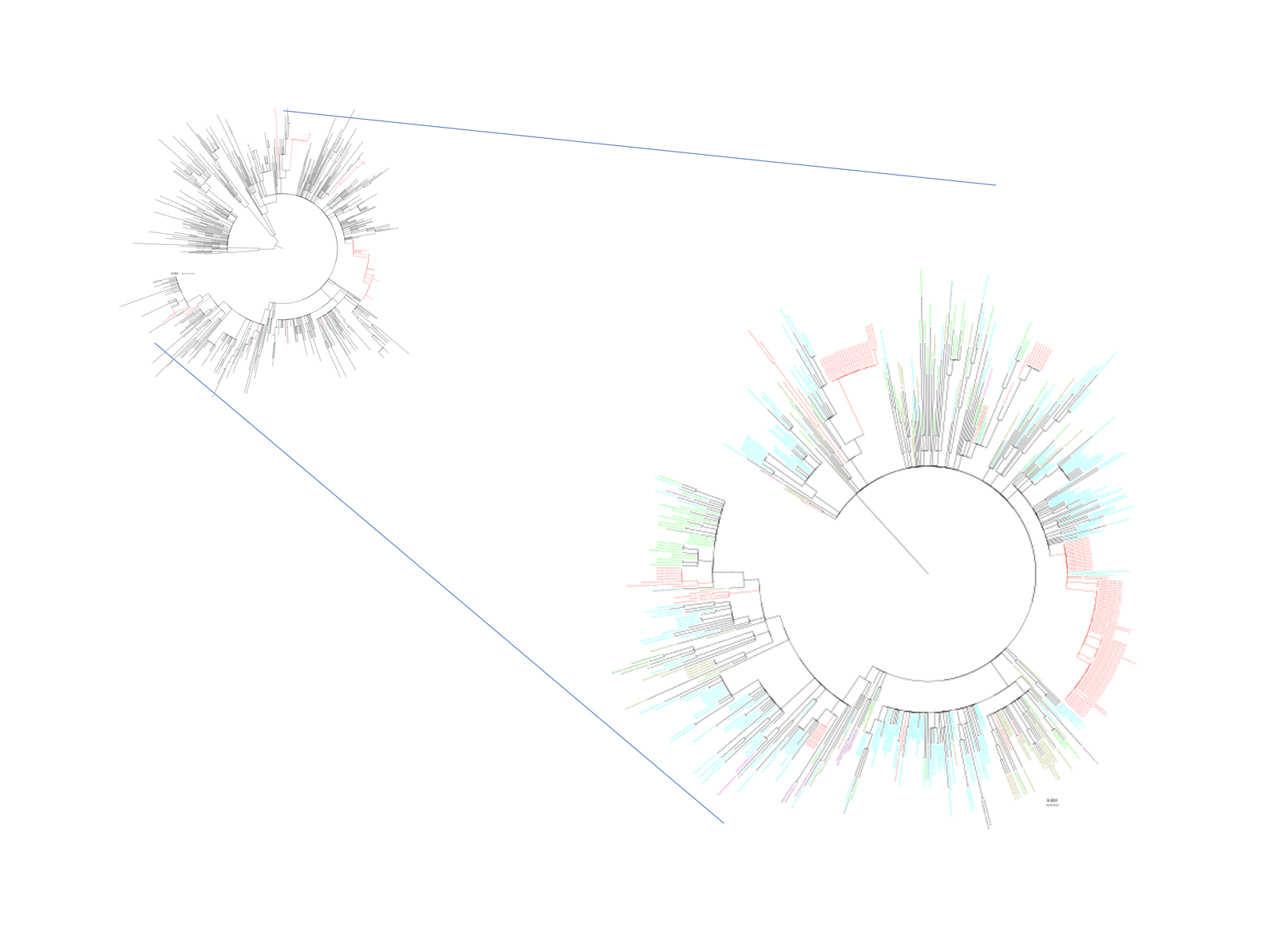


**Figure S-7:**  **Maximum-likelihood phylogenetic trees of PB1 segments of influenza A/H3N2 viruses circulating in Kamigoto and comparing sequences from strains isolated in Japan and other parts of the world from GISAID collected between 2011 and 2013. Kamigoto sequences are in red colour. The remaining strains are colored coded by region: North America in cyan, South America in teal, Oceania in green, Africa in magenta, Europe in purple, and Asia in brown.**


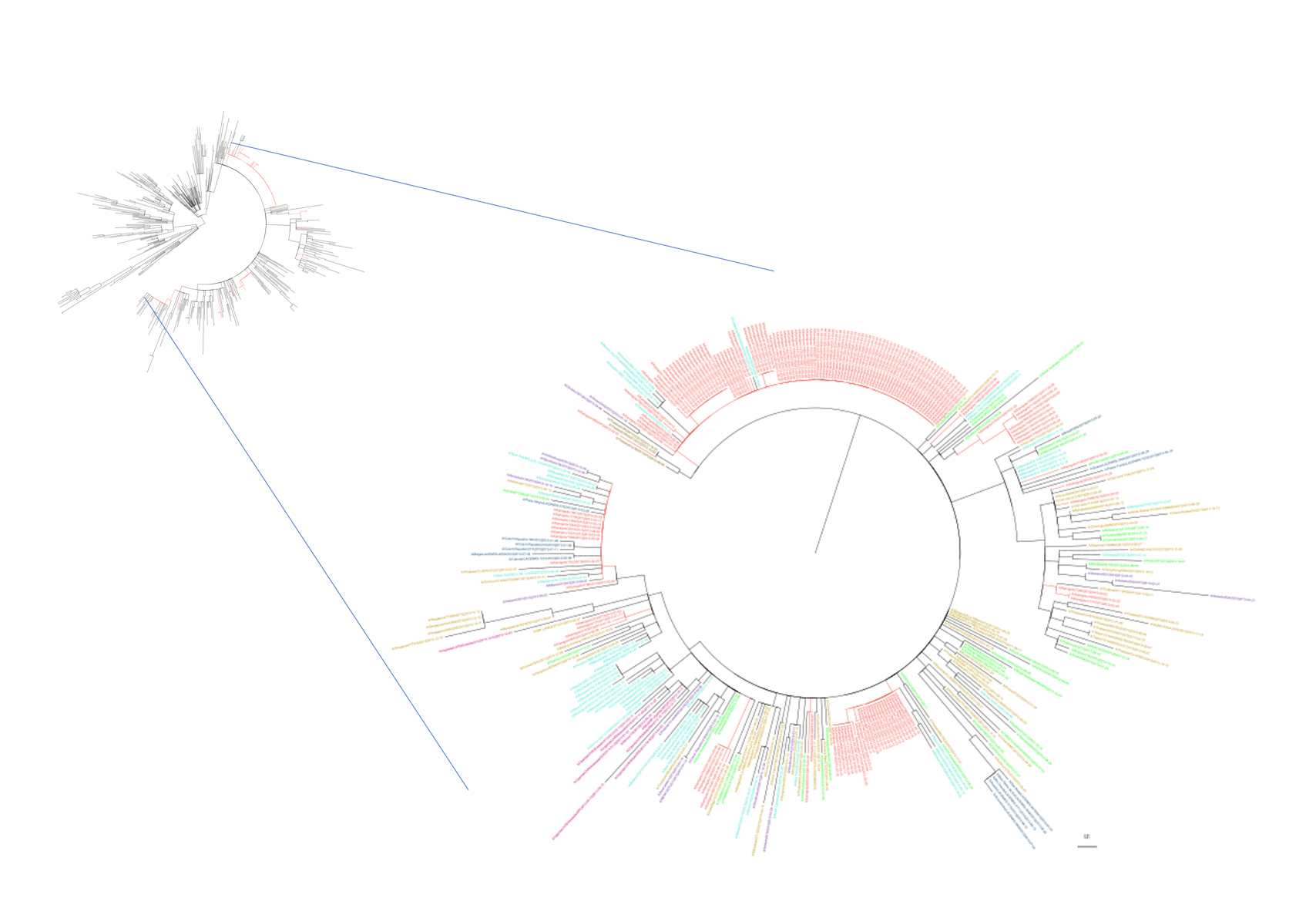


**Figure S-8:**  **Maximum-likelihood phylogenetic trees of PA segments of influenza A/H3N2 viruses circulating in Kamigoto and comparing sequences from strains isolated in Japan and other parts of the world from GISAID collected between 2011 and 2013. Kamigoto sequences are in red colour. The remaining strains are colored coded by region: North America in cyan, South America in teal, Oceania in green, Africa in magenta, Europe in purple, and Asia in brown.**

**
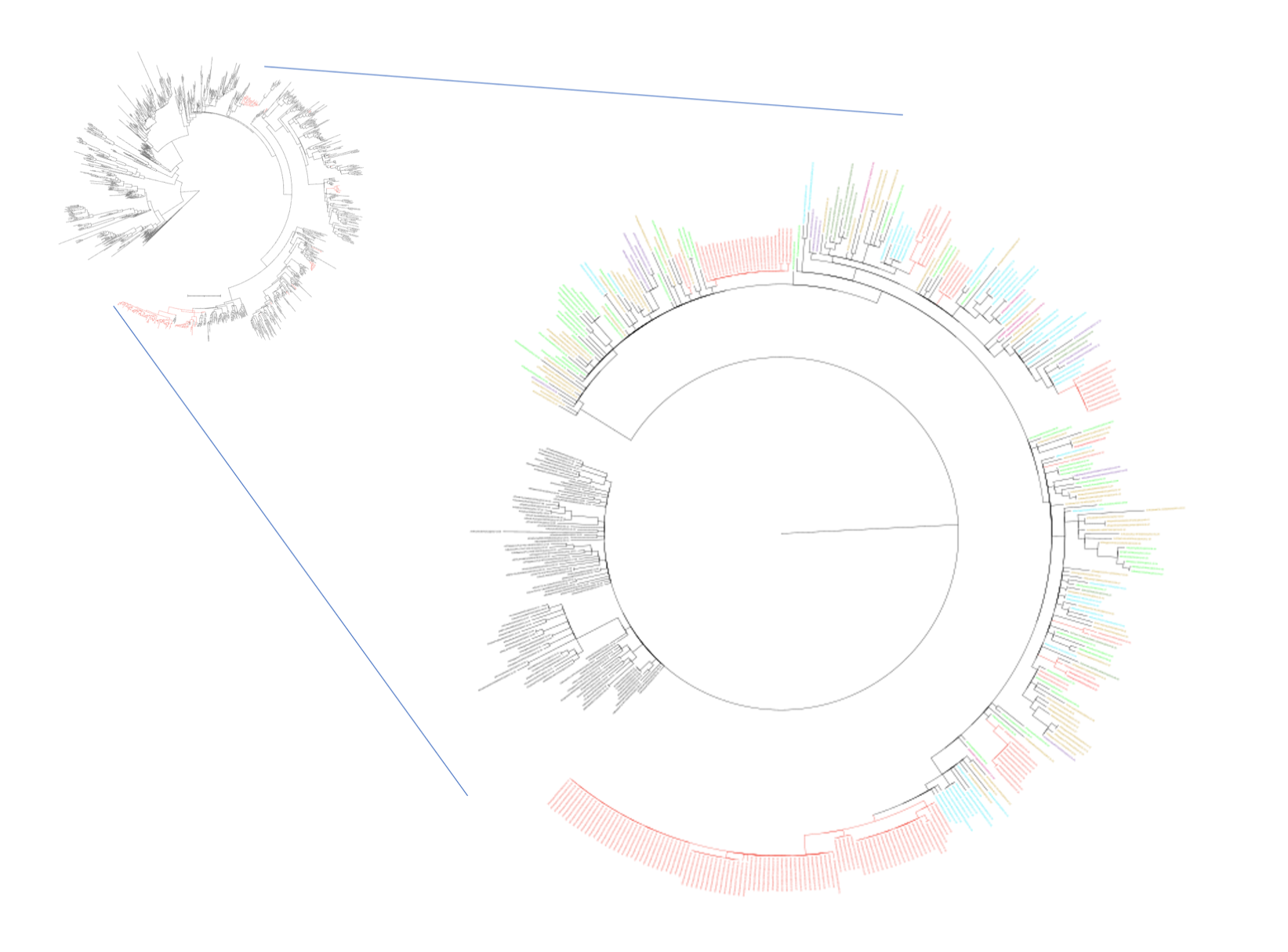
**

**Figure S-9**  **Maximum-likelihood phylogenetic trees of HA segments of influenza A/H3N2 viruses circulating in Kamigoto and comparing sequences from strains isolated in Japan and other parts of the world from GISAID collected between 2011 and 2013. Kamigoto sequences are in red colour. The remaining strains are colored coded by region: North America in cyan, South America in teal, Oceania in green, Africa in magenta, Europe in purple, and Asia in brown.**


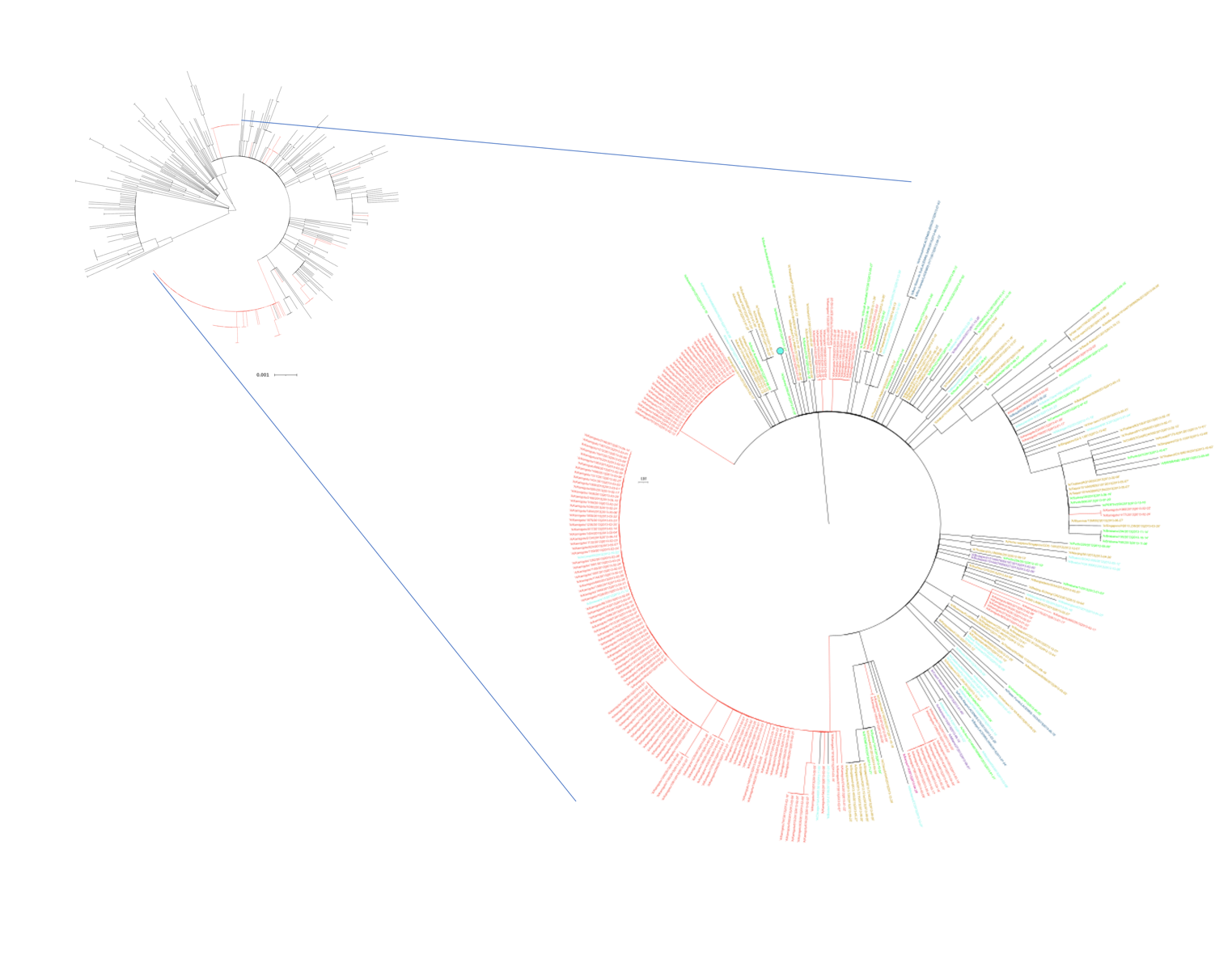


**Figure S-10:**  **Maximum-likelihood phylogenetic trees of NP segments of influenza A/H3N2 viruses circulating in Kamigoto and comparing sequences from strains isolated in Japan and other parts of the world from GISAID collected between 2011 and 2013. Kamigoto sequences are in red colour. The remaining strains are colored coded by region: North America in cyan, South America in teal, Oceania in green, Africa in magenta, Europe in purple, and Asia in brown.**

**
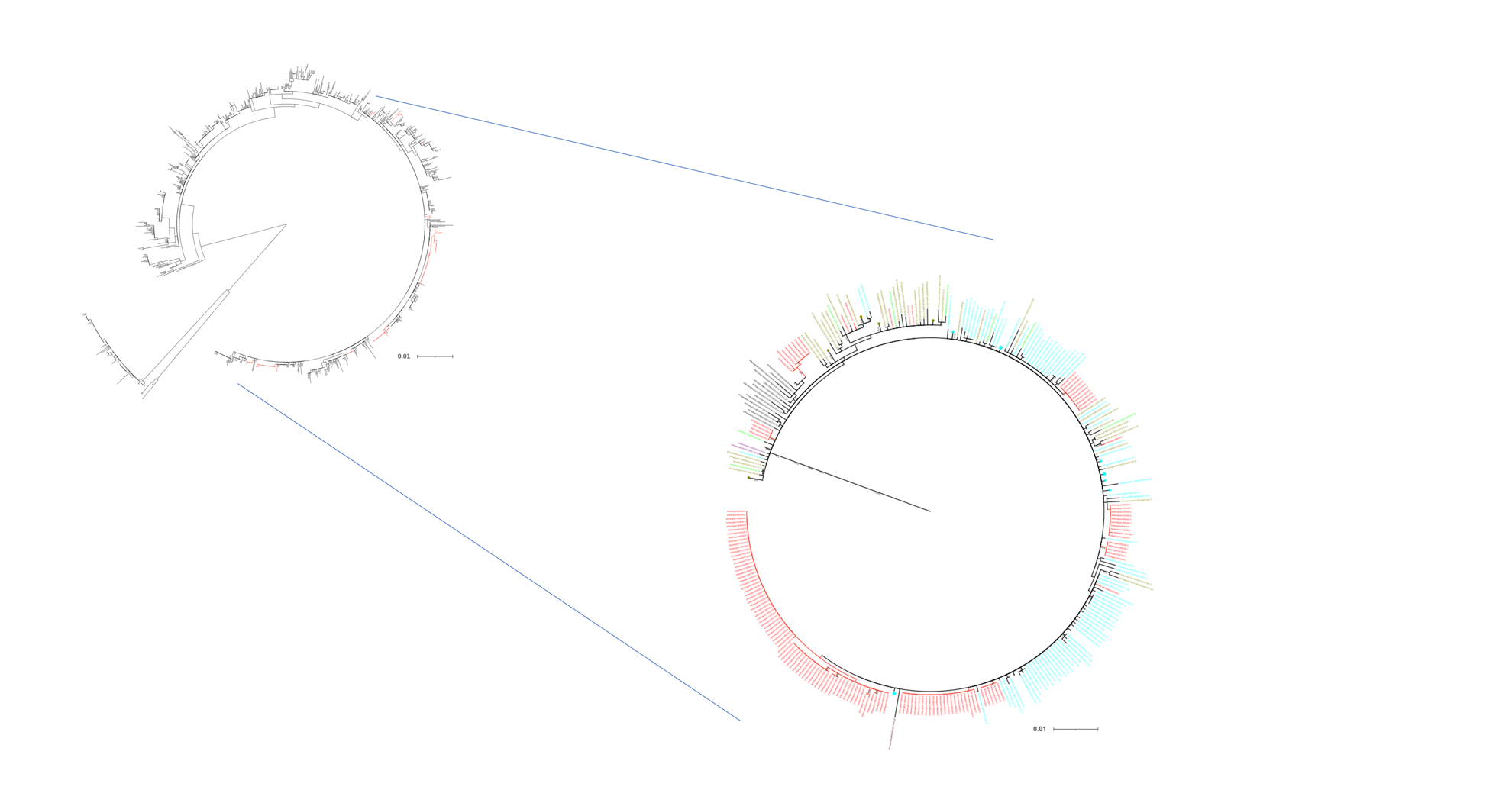
**

**Figure S-11:**  **Maximum-likelihood phylogenetic trees of NA segments of influenza A/H3N2 viruses circulating in Kamigoto and comparing sequences from strains isolated in Japan and other parts of the world from GISAID collected between 2011 and 2013. Kamigoto sequences are in red colour. The remaining strains are colored coded by region: North America in cyan, South America in teal, Oceania in green, Africa in magenta, Europe in purple, and Asia in brown.**

**
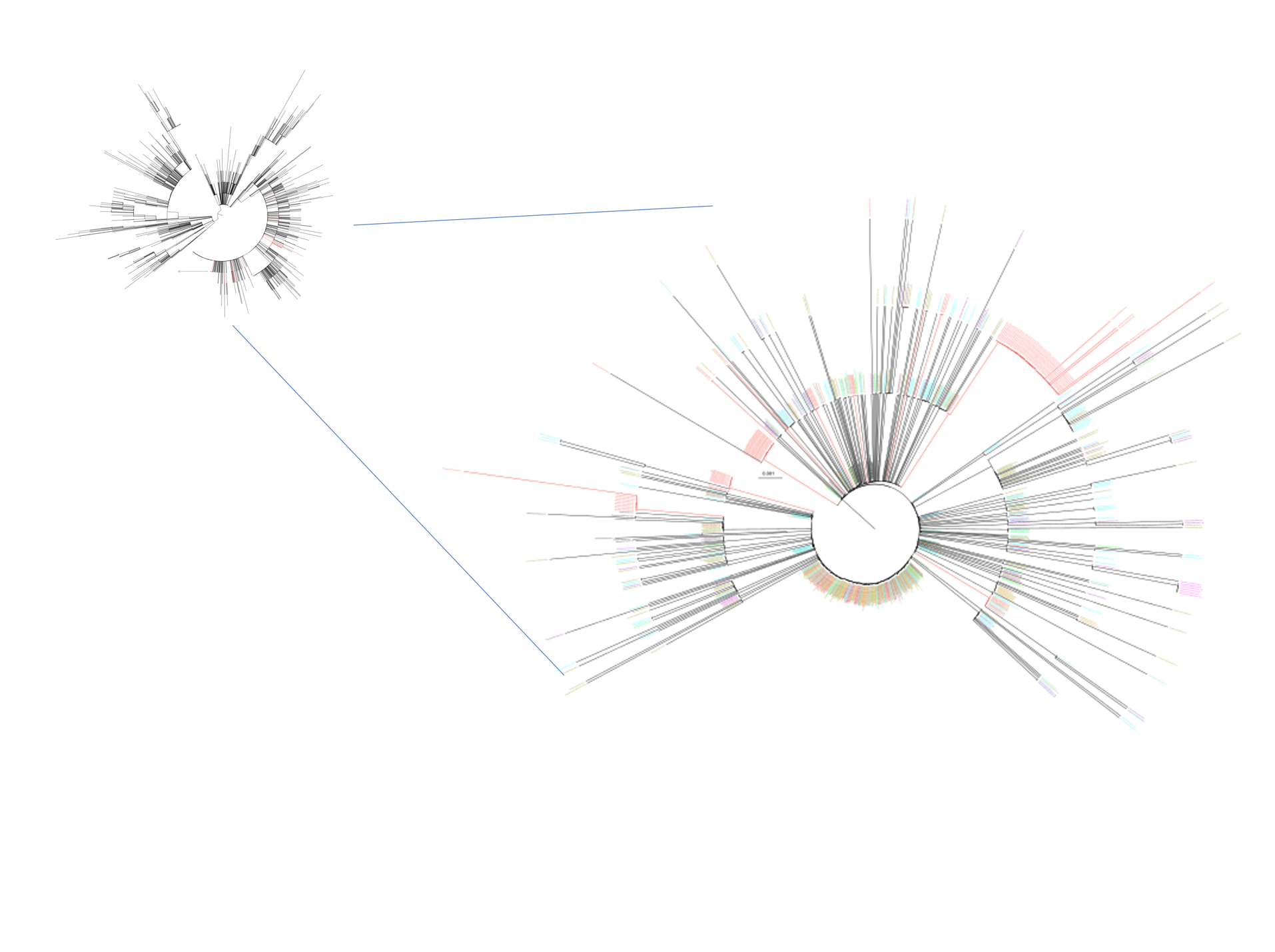
**

**Figure S-12:**  **Maximum-likelihood phylogenetic trees of MP segments of influenza A/H3N2 viruses circulating in Kamigoto and comparing sequences from strains isolated in Japan and other parts of the world from GISAID collected between 2011 and 2013. Kamigoto sequences are in red colour. The remaining strains are colored coded by region: North America in cyan, South America in teal, Oceania in green, Africa in magenta, Europe in purple, and Asia in brown.**

**
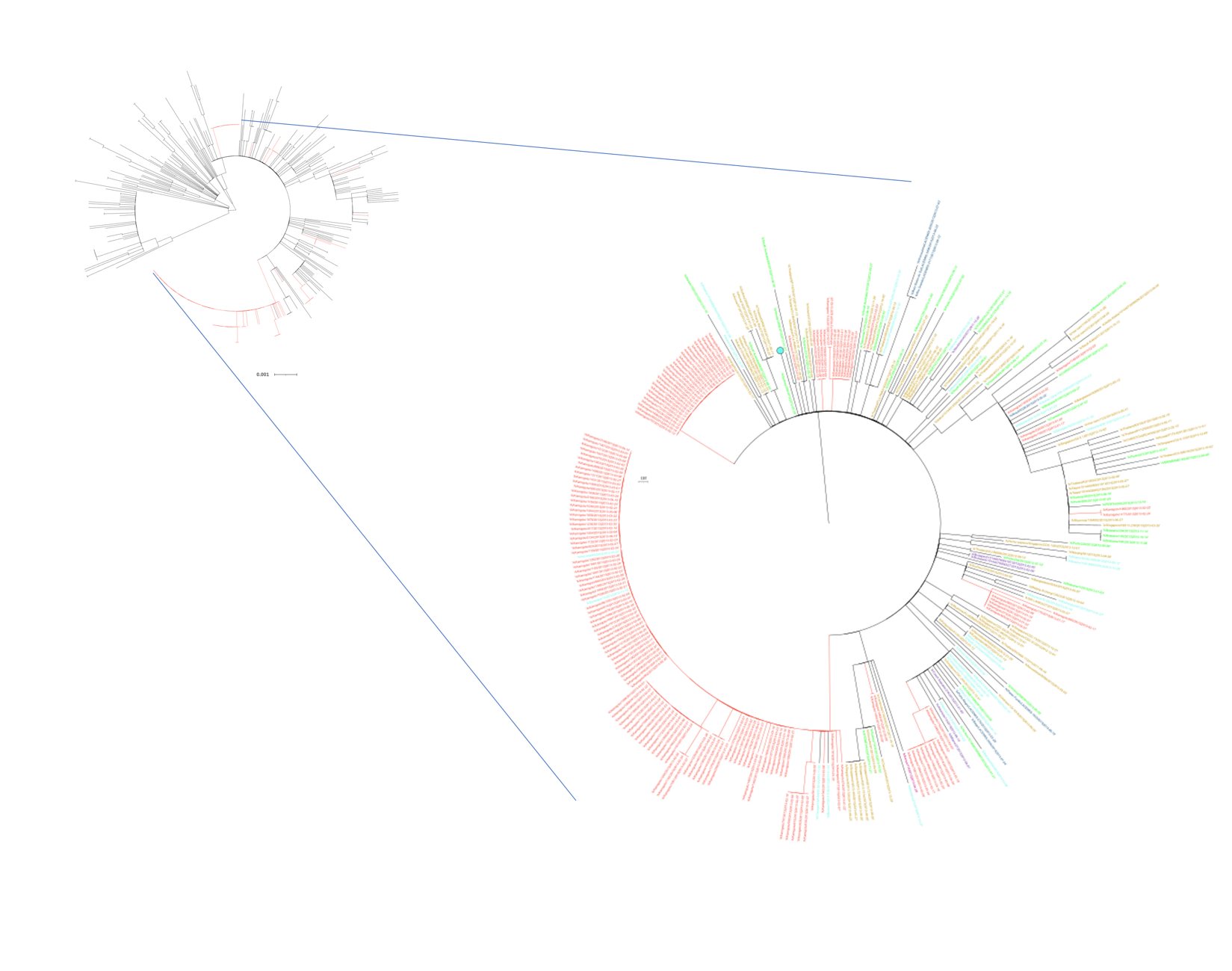
**

**Figure S-13:**  **Maximum-likelihood phylogenetic trees of NS segments of influenza A/H3N2 viruses circulating in Kamigoto and comparing sequences from strains isolated in Japan and other parts of the world from GISAID collected between 2011 and 2013. Kamigoto sequences are in red colour.**

**The remaining strains are colored coded by region: North America in cyan, South America in teal, Oceania in green, Africa in magenta, Europe in purple, and Asia in brown.**

**FIGURE S-14.** The maximum likelihood phylogenetic tree includes 166 WGS collected during the 2011/12 (red) and 2012/13 (purple) influenza seasons alongside the WGS in Japan, that were available in the GISAID (blue), and the vaccine strains (green). For each sequence the date of the sample collection is mentioned (yyyy-mm-dd). WGS for 2012/2013 in Japan were not available during the study period.

**
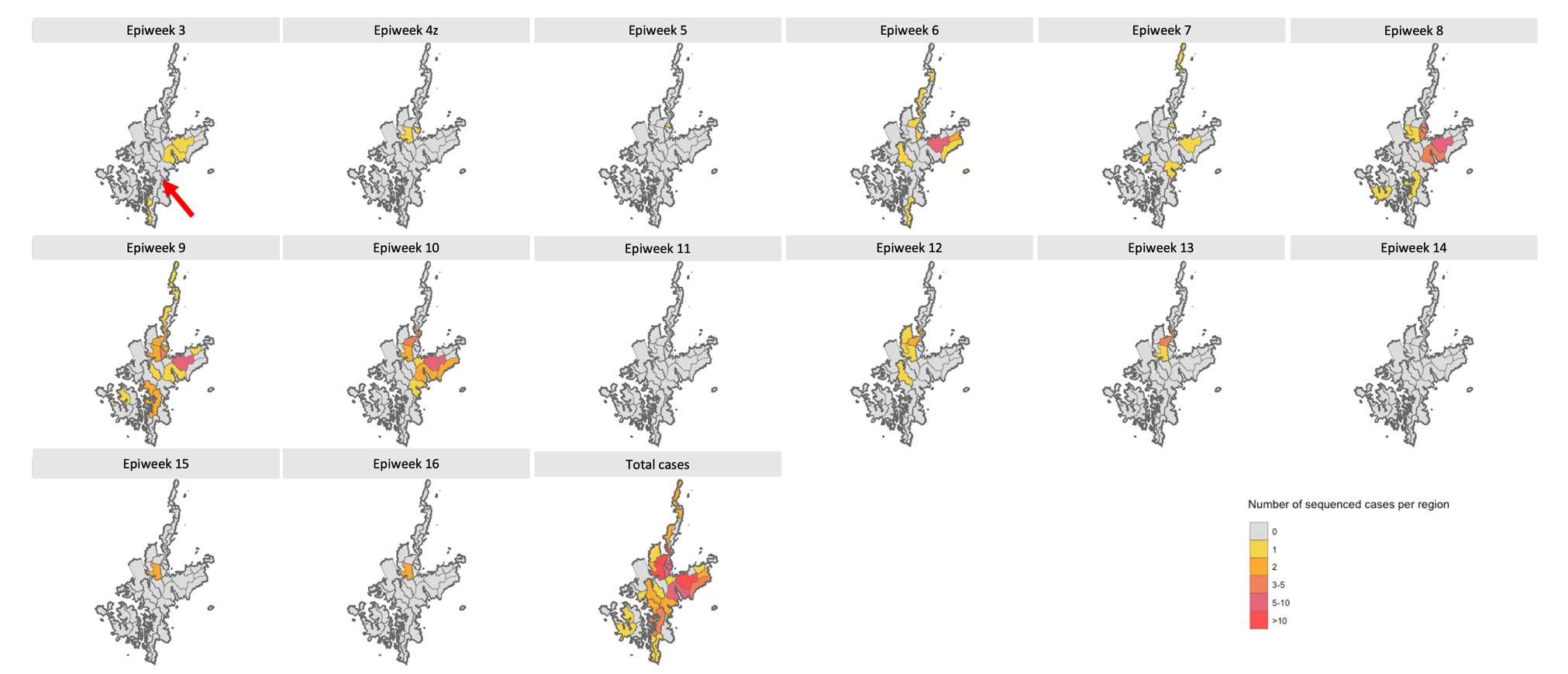
**

**Figure S-15:** Temporal and spatial distribution of the sequenced cases of cluster 5 (5A and 5B) (weekly)

**
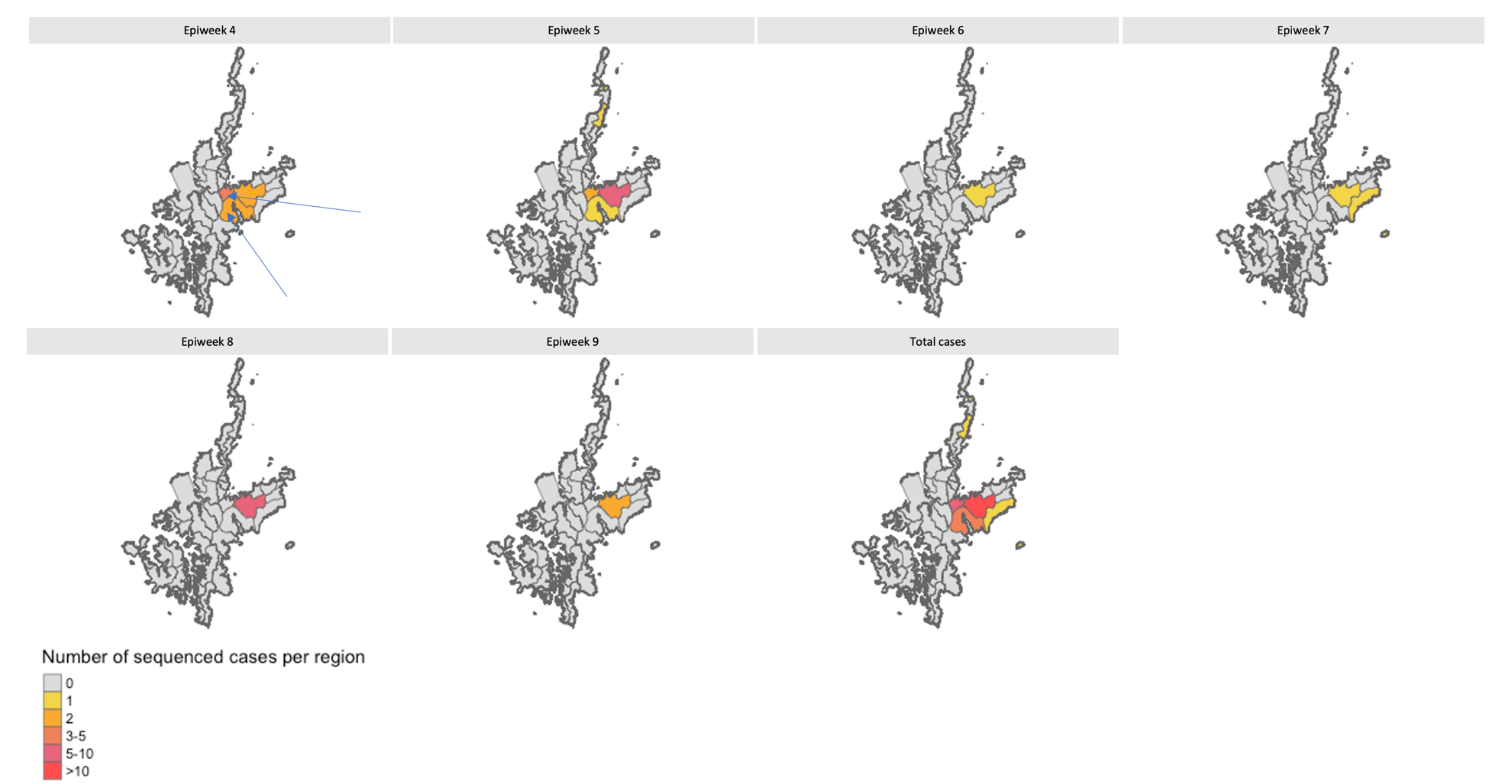
**

**Figure S-16:** Temporal and spatial distribution of the sequenced cases of cluster 1 (weekly)
